# Molecular Mechanisms Underlying Metabolic Resistance to Cyflumetofen and Bifenthrin in *Tetranychus urticae* Koch on Cowpea

**DOI:** 10.3390/ijms232416220

**Published:** 2022-12-19

**Authors:** Zhenxiu Liu, Fuxing Wu, Weikang Liang, Lijuan Zhou, Jiguang Huang

**Affiliations:** Key Laboratory of Natural Pesticide & Chemical Biology, Ministry of Education, South China Agricultural University, Guangzhou 510642, China

**Keywords:** *Tetranychus urticae*, cyflumetofen, bifenthrin, transcriptomic, metabolic resistance

## Abstract

*Tetranychus urticae* Koch (*T. urticae*) is one of the most tremendous herbivores due to its polyphagous characteristics, and is resistant to most acaricides. In this study, enzyme-linked immunosorbent assay (ELISA), transcriptome sequencing (RNA-seq) and quantitative real-time PCR (qRT-PCR) were carried out to analyze the mechanisms of *T. urticae* metabolic resistance to cyflumetofen and bifenthrin on cowpea. The enzyme activity of UDP-glucuronosyltransferases (UGTs) and carboxylesterases (CarEs) in the cyflumetofen-resistant (R_cfm) strain significantly decreased, while that of cytochrome P450 monooxygenases (P450s) significantly increased. Meanwhile, the activities of glutathione-S-transferases (GSTs), CarEs and P450s in the bifenthrin-resistant (R_bft) strain were significantly higher than those in the susceptible strain (Lab_SS). According to the Kyoto Encyclopedia of Genes and Genomes (KEGG) and Gene Ontology (GO) analyses, in the R_cfm mite strain, two carboxyl/cholinesterase (CCE) genes and two P450 genes were upregulated and one gene was downregulated, namely *CYP392E7*; in the R_bft mite strain, eleven CCE, nine UGT, two P450, four GST and three ABC genes were upregulated, while four CCE and three P450 genes were downregulated. Additionally, 94 differentially expressed genes (DEGs) were common to the two resistant groups. Specifically, *TuCCE46* and *TuCCE70* were upregulated in both resistant groups. Furthermore, the qRT-PCR validation data were consistent with those from the transcriptome sequencing analysis. Specifically, *TuCCE46* (3.37-fold) was significantly upregulated in the R_cfm strain, while in the R_bft strain, *TeturUGT22* (5.29-fold), *teturUGT58p* (1.74-fold), *CYP392A11* (2.89-fold) and *TuGSTd15* (5.12-fold) were significantly upregulated and *TuCCE01* (0.13-fold) and *CYP392A2p* (0.07-fold) were significantly downregulated. Our study indicates that *TuCCE46* might play the most important role in resistance to cyflumetofen, and *TuCCE01*, *teturUGT58p*, *teturUGT22*, *CYP392A11*, *TuGSTd15*, *TuGSTm09* and *TuABCG-13* were prominent in the resistance to bifenthrin. These findings provide further insight into the critical genes involved in the metabolic resistance of *T. urticae* to cyflumetofen and bifenthrin.

## 1. Introduction

*Tetranychus urticae* Koch (*T. urticae*), the two-spotted spider mite, is recognized as one of the most tremendous herbivores because of its polyphagous characteristic. The mite has a wide range of hosts, including more than 1400 vegetation types, among them various agricultural crops [1,2]. Yield reductions of 40–60% were reported due to the attack of *T. urticae* on soybean plants [3]. Grain losses ranging from 6–48% caused by mites have also been reported [4]. Sood et al. [5] reported the yield reduction of cucumber in different growth stages caused by mites varying from 3.2 to 62.5%. Recently, mite damage on cowpea (*Vigna unguiculata* L. Walp.) has become more and more serious [6]. Cowpeas are cultivated as an important vegetable crop in many countries. Cowpea is a grain legume that is grown extensively as an alternate protein and income source for many farmers [7,8]. Presently, the control of this mite on cowpeas is still primarily conducted using acaricides. However, the two-spotted spider mite has developed high resistance to most acaricides due to its unique traits, such as rapid development, short lifecycle, high fecundity and arrhenotokous parthenogenesis [2,9,10].

Cyflumetofen [2-methoxyethyl (*R*,*S*)-2-(4-tert-butylphenyl)-2-cyano-3-oxo-3-(*α*,*α*,*α*-trifluoro-*O*-tolyl)-propionate] is the first acaricide of complex II inhibitors which acts on the mitochondrial electron transport (MET) chain [2,11,12,13,14]. This acaricide is a pro-pesticide and can form an active de-esterified metabolite to decrease the activity of succinate dehydrogenase and promote the oxidation process of succinate to fumarate in the Krebs cycle [2,15]. Recently, studies have shown that increased detoxification is pertinent to the resistance of the two-spotted spider mite to cyflumetofen [14]. Khalighi et al. [15] reported that glutathione-S-transferases (GSTs) may contribute to resistance to cyflumetofen, and thus the synergists piperonyl butoxide (PBO) and S,S,S-tributyl phosphorotrithioate (DEF) did not enhance the toxicity of cyflumetofen. Furthermore, Pavlidi et al. [12] demonstrated that GSTd05 could directly metabolize cyflumetofen. Recently, Sugimoto et al. [16] reported that *TuGSTd05* in the SoOm1_CflR strain of *T. urticae* was upregulated. Moreover, downregulated carboxyl/cholinesterase (CCE) genes were also related to cyflumetofen resistance [16]. Bifenthrin, a broad-spectrum pyrethroid acaricide, plays an important role in the control of *T. urticae* [17,18]. The metabolic mechanisms of pyrethroid resistance have been increasingly studied since the first report of the bifenthrin resistance of *T. urticae* in 1992 [19]. Of these resistance mechanisms, cytochrome P450 monooxygenases (P450s) and carboxylesterases (CarEs) are crucial for increasing metabolic detoxification, while glutathione-S-transferase exerts a negligible influence on pyrethroid resistance [16,19,20]. Ay and Gürkan [20] found that esterase activity was closely related to the high resistance to bifenthrin in *T. urticae*. In addition, Van Leeuwen et al. [19] demonstrated that the toxicity of bifenthrin was obviously promoted when adding the esterase inhibitor DEF.

Overall, the specific metabolic resistance mechanism is still unclear in *T. urticae*, though there are various studies addressing the metabolic resistance mechanism and the detoxification genes related to cyflumetofen and bifenthrin [14,19,21,22,23]. Therefore, we selected cyflumetofen- and bifenthrin-resistant strains from the same parent strain (a colony reared in the laboratory for more than 6 years without exposure to any acaricide) and then performed enzyme activity assays by using the enzyme-linked immunosorbent assay (ELISA) method, and transcriptome sequencing (RNA-seq) together with validation by quantitative real-time PCR (qRT-PCR) to explore their metabolic resistance mechanism.

## 2. Results

### 2.1. Selection of the Cyflumetofen- and Bifenthrin-Resistant Strains

The initial median lethal concentration (LC_50_) values of the strain to cyflumetofen and bifenthrin were 6.02 and 125.04 mg/L, respectively. After 16 generations of selection by spraying with cyflumetofen, the LC_50_ value of the cyflumetofen-resistant (R_cfm) strain was 707.95 mg/L, and its resistance ratio (RR) was 117.60. After spraying with bifenthrin for 20 consecutive generations, the LC_50_ value of the bifenthrin-resistant (R_bft) strain was higher than 20000 mg/L, and the RR was greater than 159.95 (Table 1).

### 2.2. The Enzyme Activity in the Cyflumetofen- and Bifenthrin-Resistant Strains

**Figure 1:**
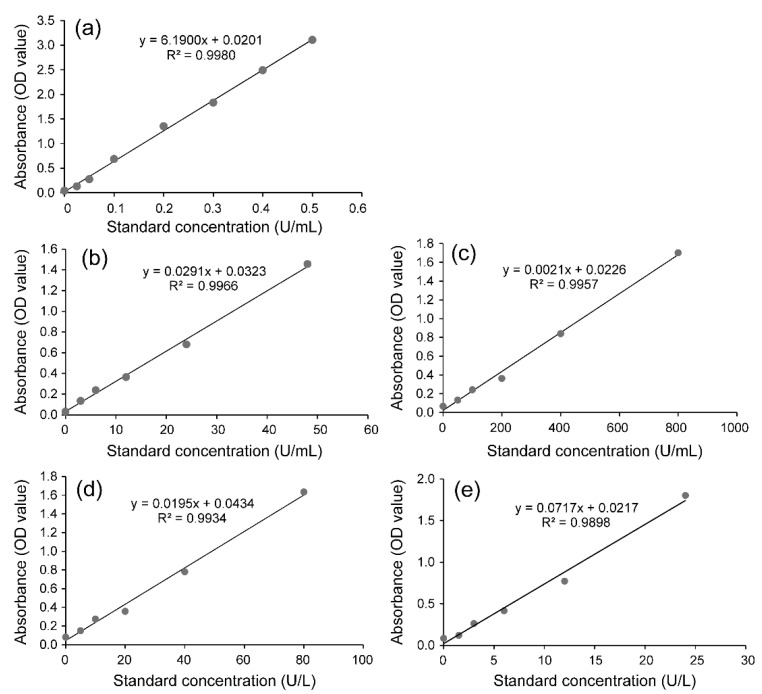
The standard curves of the content of protein (**a**), UDP-glucuronosyltransferases (UGTs) (**b**), carboxylesterases (CarEs) (**c**), glutathione-S-transferases (GSTs) (**d**) and cytochrome P450 monooxygenases (P450s) (**e**).

The standard curve of the content of protein was y = 6.1900x + 0.0201. The correlation coefficient (R^2^) was 0.9980 (Figure 1a). Based on this, the standard curves of the contents of UGTs, CarEs, GSTs and P450s were determined (Figure 1b–e). 

**Figure 2:**
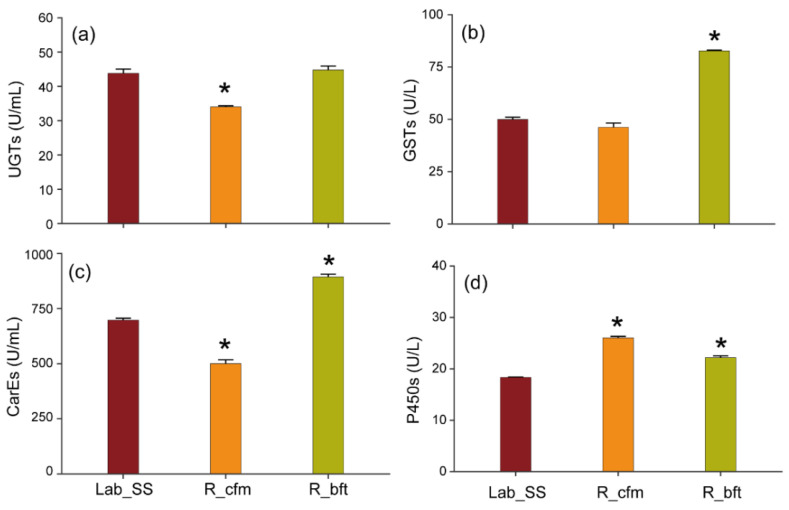
The enzyme activity in the cyflumetofen- (R_cfm) and bifenthrin-resistant (R_bft) strains of *T. urticae*. The activity of UDP-glycosyltransferases (UGTs) (**a**), glutathione-S-transferases (GSTs) (**b**), carboxylesterases (CarEs) (**c**) and cytochrome P450 monooxygenases (P450s) (**d**). * indicates the significant difference (*p* < 0.05) compared with the susceptible strain (Lab_SS).

Compared with the susceptible strain (Lab_SS), the activities of UGTs and CarEs in the cyflumetofen-resistant strain were significantly decreased, with activity values of 34.02 and 500.52 U/mL, respectively (*p* < 0.05, Figure 2a,c). In contrast, the activity of P450s was significantly increased and the value was 26.01 U/L (*p* < 0.05, Figure 2d). However, the activities of GSTs, CarEs and P450s in the bifenthrin-resistant strain were significantly higher than those in the susceptible strain and the activity values were 82.71 U/L, 893.69 U/mL and 22.19 U/L, respectively (*p* < 0.05, Figure 2b–d).

### 2.3. Transcriptome Sequencing, Data Processing and Differential Gene Expression Analysis

A comparative transcriptome analysis of Lab_SS vs R_bft and Lab_SS vs R_cfm was used to elucidate the patterns of the gene expression. Six cDNA libraries were prepared for transcriptome sequencing (BGISEQ-50 platform). In total, 127.33 million clean reads for the Lab_SS strain, 124.72 million clean reads for the R_cfm strain and 128.13 million clean reads for R_bft strain were obtained. The values of Q20 and Q30 were more than 94% and 88%, respectively, denoting the sufficiency of the data in the transcriptome analysis (Table 2).

**Figure 3:**
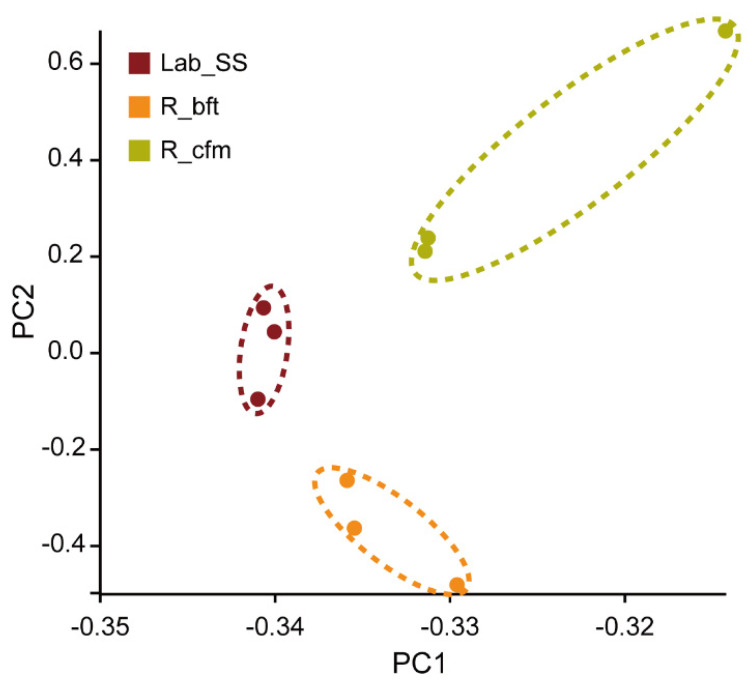
PCA plots of the susceptible strain (Lab_SS), cyflumetofen- (R_cfm) and bifenthrin-resistant (R_bft) strains of *T. urticae*. Different colours represent different treatments (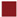:Lab_SS; 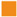: R_bft; 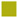: R_cfm).

**Figure 4:**
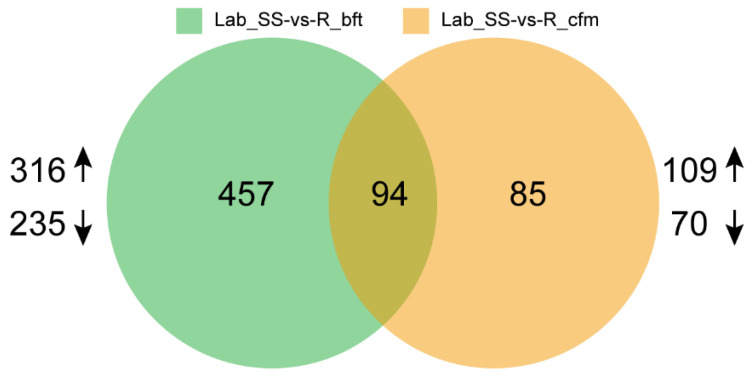
Summary of differentially expressed genes between the Lab_SS vs R_cfm group and the Lab_SS vs R_bft group.

Furthermore, the principal component analysis (PCA) indicated that the gene expression in the R_cfm and R_bft strains obviously differed from that of the Lab_SS strain (Figure 3). Then, 551 and 179 differentially expressed genes (DEGs) were filtered out in the groups of Lab_SS vs R_bft and Lab_SS vs R_cfm, respectively, based on the criteria of |log_2_FC| ≥ 1 and FDR < 0.05 in the differential expression (DE) analysis (Figure 4).

For the Lab_SS vs R_cfm group, 109 genes were upregulated (fold change ≥ 2.00 and adjusted *p* value < 0.05) and 70 genes were downregulated, which was presented in the scatter plot showing the distribution of the DEGs (Figure S1a,b). Similarly, For the Lab_SS vs R_bft group, 316 genes were upregulated (fold change ≥ 2.00 and adjusted *p* value < 0.05) and 235 genes were downregulated, which is presented in the scatter plot showing the distribution of the DEGs (Figure S1c,d).

### 2.4. Functional Annotation

**Figure 5:**
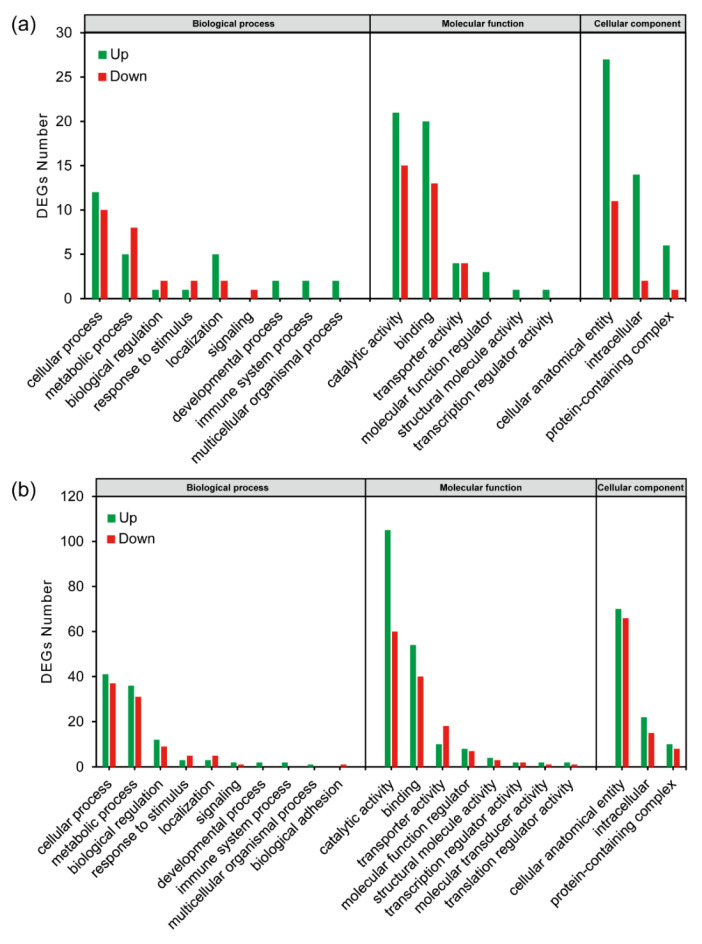
Gene ontology enrichment analysis of the differentially expressed genes from the cyflumetofen-resistant (R_cfm) (**a**) and the bifenthrin-resistant (R_bft) (**b**) strains of *T. urticae*.

The DEGs were classified by Gene Ontology (GO) analysis. With the threshold of |log_2_FC| ≥ 1 and FDR < 0.05, 78 and 299 genes that were significantly differentially expressed were annotated in Lab_SS vs R_cfm and Lab_SS vs R_bft, respectively (Figure 5a,b). 

#### 2.4.1. Functional Annotation of the Lab_SS vs R_cfm Group

For the Lab_SS vs R_cfm group, the results indicated that the DEGs could be associated with 15 biological processes, 3 cellular components and 13 molecular functions (Table S1). There were 15 significantly enriched GO terms in the biological process category. Among them, four significantly enriched GO terms (DNA integration, DNA metabolic process, autophagy and process utilizing autophagic mechanism) accounted for 19.23%, 23.08%, 11.54% and 11.54%, respectively. In the cellular component category, three GO terms (nucleosome, DNA packaging complex and protein-DNA complex) were significantly enriched and accounted for the same rate of 7.89%. Furthermore, in the molecular function category, 13 GO terms were significantly enriched. In this category, endopeptidase activity, peptidase activity and peptidase activity (acting on L-amino acid peptides) were significantly enriched and accounted for 15.38%, 20.00% and 16.92%, respectively (Table S1). 

#### 2.4.2. Functional Annotation of the Lab_SS vs R_bft Group

For the Lab_SS vs R_bft group, the results indicated that the DEGs could be related to 21 biological processes, 2 cellular components and 10 molecular functions (Table S2). There were 21 GO terms significantly enriched in the biological process category. Among them, four significantly enriched GO terms accounted for more than 10.00%. They were sphingolipid metabolic process (10.48%), membrane lipid metabolic process (11.43%), cellular lipid metabolic process (16.19%) and lipid metabolic process (20.00%). However, in the cellular component category, only two GO terms were significantly enriched, which were the integral component of the membrane and intrinsic component of the membrane, respectively, and each of them accounted for the same rate of 72.46%. Meanwhile, in the molecular function category, 10 GO terms were significantly enriched. Of these 10 GO terms, four of them accounted for more than 10.00%. They were catalytic activity (67.35%), peptidase activity (13.88%), hydrolase activity (34.29%) and peptidase activity, acting on L-amino acid peptides (11.84%) (Table S2). 

Overall, the multiple biological processes involved in the DEGs, the functional variety of the cellular components and the variety of molecular functions indicate that the resistance mechanism of the mites to cyflumetofen or bifenthrin is related to complicated physiological and biochemical processes.

#### 2.4.3. Functional Annotation of the Common DEGs between the Lab_SS vs R_cfm Group and the Lab_SS vs R_bft Group

**Figure 6:**
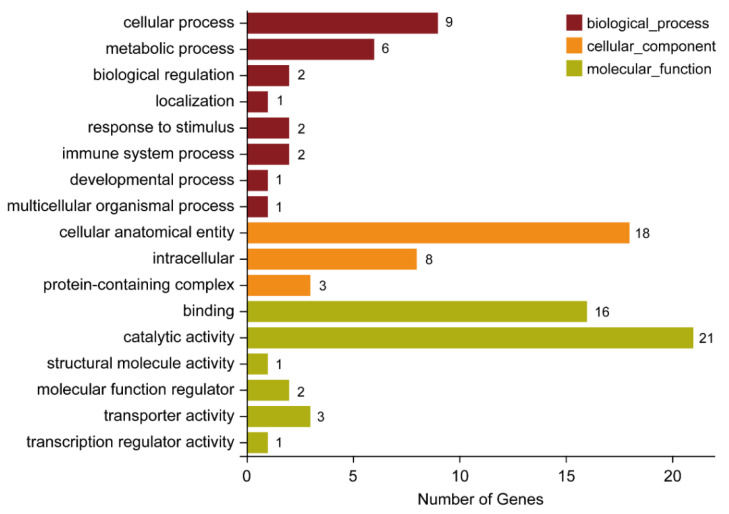
Gene ontology analysis of the differentially expressed genes common to the Lab_SS vs R_cfm group and the Lab_SS vs R_bft group.

The results in Figure 4 indicated that the number of the common DEGs between the two groups (the Lab_SS vs R_cfm group and the Lab_SS vs R_bft group) was 94 (Figure 4). Among these 94 DEGs, 69 of them were significantly enriched, which could be associated with 58 biological processes, 6 molecular functions and 3 cellular components (Table S3). And among these 94 DEGs, 45 of them were classified and annotated. In the biological process category, the common DEGs of the two groups were primarily associated with cellular process and metabolic process. In detail, 9 DEGs were associated with the cellular process, while 6 DEGs were associated with the metabolic process. Regarding the molecular functions category, the common DEGs of the two groups were mainly associated with binding and catalytic activity. In detail, 16 DEGs were associated with binding, while 21 DEGs were associated with catalytic activity. In the cellular components category, the number of the common DEGs was 18 and they were associated with a cellular anatomical entity (Figure 6).

### 2.5. Kyoto Encyclopedia of Genes and Genomes (KEGG) Pathway Analysis

With the threshold of |log_2_FC| ≥ 1 and FDR < 0.05, the top 20 biological metabolic pathways were obtained in the KEGG database (Figure S2a,b).

#### 2.5.1. KEGG Pathway Analysis of the Lab_SS vs R_cfm Group

**Figure 7:**
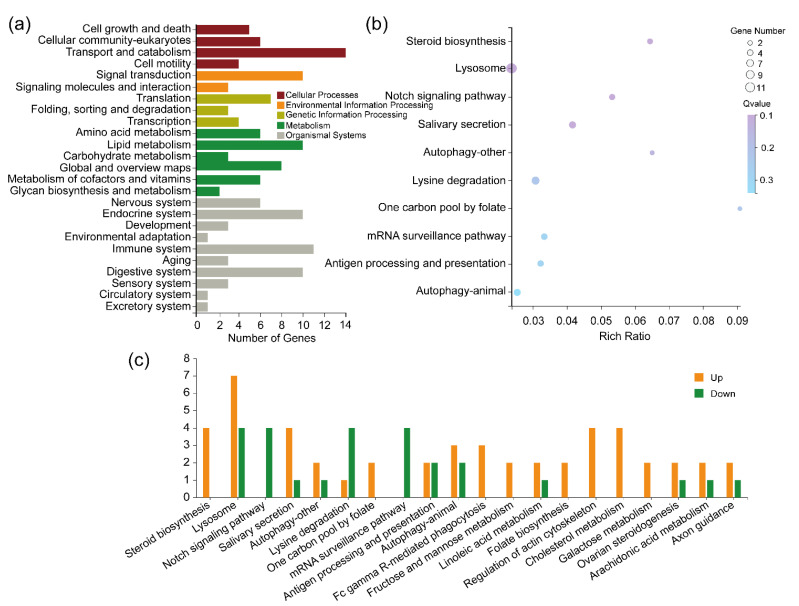
Kyoto Encyclopedia of Genes and Genomes (KEGG) analysis of differentially expressed genes from the cyflumetofen-resistant (R_cfm) strain of *T. urticae*. (**a**) Classified KEGG pathways; (**b**) enriched KEGG pathways; (**c**) the number of differently expressed genes in the most enriched pathways.

Regarding the biological functions of DEGs in the cyflumetofen-resistant strain, the KEGG enrichment analysis showed that there were 6770 annotated genes in the detected metabolic pathways (Table S4). Compared to the susceptible strain, in the cyflumetofen-resistant strain, 64 DEGs were annotated, which were primarily associated with five major metabolic pathways: cellular processes, environmental information processing, genetic information processing, metabolism and organismal systems (Table S4, Figure 7a). There were 10 significantly enriched pathways in the 87 metabolic pathways (Figure 7b). The up- and downregulated DEGs in the top 20 metabolic pathways are presented in Figure 7c. This enrichment is presented in Table S4 and indicated that these 10 significantly enriched pathways might be important for mite resistance to cyflumetofen.

#### 2.5.2. KEGG Pathway Analysis of the Lab_SS vs R_bft Group

**Figure 8:**
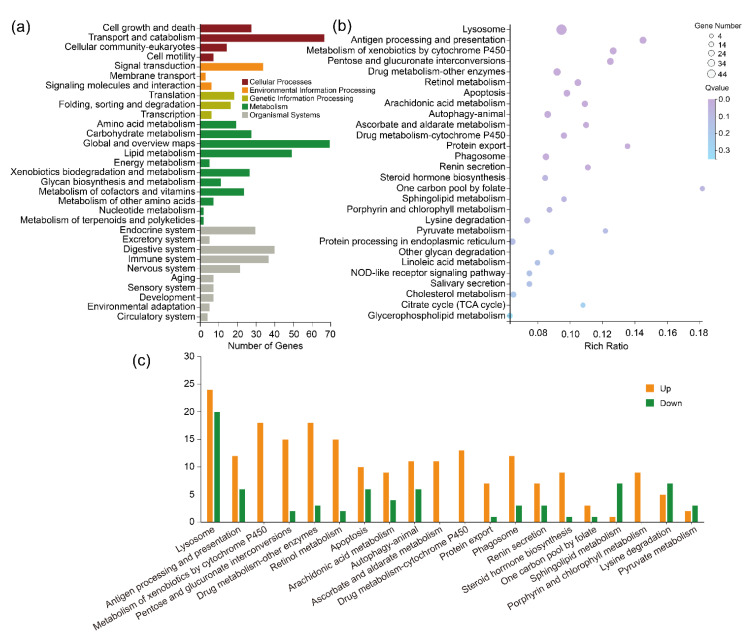
Kyoto Encyclopedia of Genes and Genomes (KEGG) analysis of differentially expressed genes from the bifenthrin-resistant (R_bft) strain of *T. urticae*; (**a**) classified KEGG pathways; (**b**) enriched KEGG pathways; (**c**) the number of differently expressed genes in the most enriched pathways.

Regarding the biological functions of DEGs in the bifenthrin-resistant strain, the KEGG enrichment analysis showed that there were 6770 annotated genes in the whole of the detected metabolic pathways (Table S5). Compared to the susceptible strain, in the bifenthrin-resistant strain, 224 DEGs were annotated and majority of them were related to five major metabolic pathways: cellular processes, environmental information processing, genetic information processing, metabolism and organismal systems (Table S5, Figure 8a). It was shown that 28 of the 189 metabolic pathways were significantly enriched (Figure 8b). The up- and downregulated DEGs in the top 20 metabolic pathways are presented in Figure 8c. This enrichment was shown in Table S5 and indicated that these 28 significantly enriched pathways might be important for mite resistance to bifenthrin.

#### 2.5.3. KEGG Pathway Analysis of the Common DEGs between the Lab_SS vs R_cfm Group and the Lab_SS vs R_bft Group

The KEGG enrichment analysis revealed that 34 of the 94 DEGs common to the two groups (the Lab_SS vs R_cfm group and the Lab_SS vs R_bft group) were annotated and they could be associated with 53 metabolic pathways, and they were primarily classified into five major metabolic pathways: cellular processes, environmental information processing, genetic information processing, metabolism and organismal systems. The up- and downregulated DEGs in the top 20 metabolic pathways are presented in Figure S3. This enrichment was shown in Table S6 and indicated that these 20 significantly enriched pathways might be important for mite resistance, both to cyflumetofen and bifenthrin.

### 2.6. Selected DEGs of Detoxification Enzymes of Lab_SS vs R_cfm and Lab_SS vs R_bft

Combined with the result of the GO analysis, five detoxification genes were obtained in the cyflumetofen-resistant strain compared with the susceptible population. Of these genes, two CCE genes (*TuCCE46* and *TuCCE70*) and two P450 genes (*CYP392A2p* and *CYP392A1*) were upregulated. The upregulation fold changes of these four genes were 1.77, 1.28, 1.64 and 1.10, respectively. Additionally, *CYP392E7* was downregulated (Table 3). 

In the bifenthrin-resistant strain, 29 detoxification genes were identified. Among the upregulated genes in the bifenthrin-resistant strain, there were eleven CCE, nine UGT, two P450, four GST and three ABC genes. In detail, the upregulation fold changes of *TuCCE46* and *TuCCE70* were 3.68 and 3.09, respectively. The upregulation fold changes of *CCEincTu16*, *TuCCE45*, *TuCCE44* and *CCEincTu08* varied from 2.04 to 2.65. The upregulation fold changes of *teturUGT16*, *teturUGT22* and *teturUGT58p* were 3.31, 2.83 and 2.29, respectively. Additionally, the upregulation fold change of *CYP392D2* was 2.04. The upregulation fold changes of the other 29 genes ranged from 1.06 to 1.89 (Table 3).

Meanwhile, seven other genes of detoxification enzymes, including four CCE genes (*TuCCE40*, *TuCCE22*, *TuCCE61* and *TuCCE01*) and three P450 genes (*CYP392D7*, *CYP385C4v2* and *CYP392A2p*), were downregulated (Table 3).

### 2.7. Validation of Detoxification Enzyme Genes

Furthermore, five genes of the corresponding detoxification enzymes of the mite strain of Lab_SS vs R_cfm and 16 genes of the corresponding detoxification enzymes of the mite strain of Lab_SS vs R_bft were verified by qPCR.

**Figure 9:**
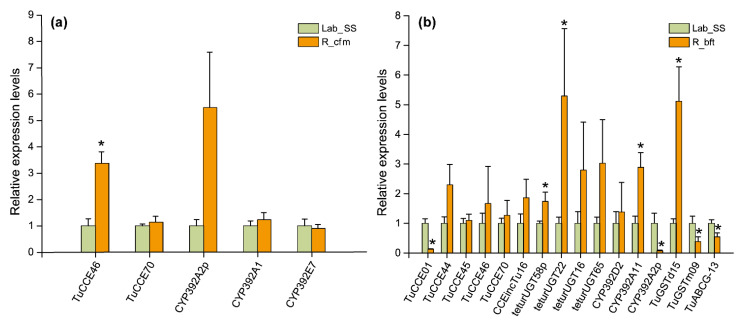
Validation of the transcriptome sequencing results by quantitative real-time PCR. (**a**) Comparison between the susceptible strain (Lab_SS) and the cyflumetofen-resistant strain (R_cfm) of *T. urticae*. (**b**) Comparison between the Lab_SS strain and the bifenthrin-resistant (R_bft) strain of *T. urticae*. * indicates the significant difference (*p* < 0.05) compared with the Lab_SS.

In the strain of Lab_SS vs R_cfm, the trends of variation of *TuCCE46*, *TuCCE70*, *CYP392A2p*, *CYP392A1* and *CYP392E7* were consistent with the results of the RNA-seq analysis (Figure 9a). Of these genes, *TuCCE46* was significantly upregulated, with 3.37-fold higher expression than in the susceptible population. 

In the strain of Lab_SS vs R_bft, the qRT-PCR data were almost consistent with the RNA-seq analysis (Figure 9b). Four genes, including *teturUGT22*, *teturUGT58p*, *CYP392A11* and *TuGSTd15*, were significantly upregulated, whose expressions were 5.29-, 1.74-, 2.89- and 5.12-fold higher than in the susceptible strain, respectively. *TuCCE01* and *CYP392A2p* were significantly downregulated by 0.13- and 0.07-fold compared to the susceptible strain, respectively. *TuCCE46* and *TuCCE70* were 1.67- and 1.27-fold higher expression than in the susceptible population, respectively. By contrast, the upregulated *TuGSTm09* and *TuABCG-13* in the RNA-seq analysis were significantly downregulated by 0.39- and 0.55-fold compared to the susceptible strain, respectively.

## 3. Discussion

From our results, we concluded that the increased P450 monooxygenase activity together with the decreased activities of UGTs and esterases were related to the high-level resistance of the mites to cyflumetofen in the enzymatic activity assays in vitro. Khalighi et al. [15] found that piperonyl butoxide (PBO, inhibitor of P450s) and S,S,S-tributyl phosphorotrithioate (DEF, inhibitor of CCEs) synergized cyflumetofen toxicity in the resistant mite strain TU008R. Similarly, Feng et al. [24] reported that the increased activities of P450s and GSTs were related to cyflumetofen resistance. Our study was in accordance with previous studies [16,22].

Previous studies reported that the detoxification of bifenthrin in *T. urticae* was associated with increased esterase activity rather than cytochrome P450s [15,16]. However, in our study, the activity of P450s, GSTs and CarEs increased and could thus play a vital role in bifenthrin resisitance [17,25]. Moreover, the activity of GSTs (R_bft) was significantly higher than that of the susceptible strain (Lab_SS) (Figure 1b), which is in agreement with the work described by Yang et al. in 2001 [26].

Carboxyl/cholinesterases (CCEs) participate in various metabolic reactions, including neurogenesis, xenobiotic detoxification, pheromone degradation and developmental regulation, and are thus considered major detoxification genes [14,27,28]. They play a critical role in the detoxification of commonly used pesticides including organophosphates, pyrethroids and carbamates through isolation and direct metabolism [29]. Previous studies confirmed that both upregulated and downregulated CCE genes contribute to cyflumetofen resistance [14,30]. In our study, *TuCCE46* was significantly upregulated in the cyflumetofen resistant strain (R_cfm) (Figure 9a). Therefore, *TuCCE46* may be pertinent to a specific upregulated esterase that could detoxify cyflumetofen and its intermediate metabolic substance [14]. However, it is yet to be verified whether the upregulated CCE genes only sequester instead of metabolizing. In addition, five upregulated CCE genes were found in the bifenthrin resistant strain (R_bft) (Figure 9b), of which upregulated *TuCCE45* has been reported in a multi-resistance population [22]. Therefore, we speculated that these genes may contribute to bifenthrin resistance.

UDP-glucosyltransferases (UGTs), a gene family of glycosyltransferases, can catalyze the binding of various small lipophilic molecules to uridine diphosphate glucose (UDPG) and increase their solubility in water [31]. Therefore, glycosylation of UGTs plays a crucial role not only in the detoxification of exogenous substances but also in the biosynthesis, storage and transportation of secondary metabolites [32,33]. Vertebrate UGTs, as critical phase II enzymes, participate in endo- and xenobiotic metabolisms in detoxification and elimination [34]. In insects, compared to other enzymes such as P450s, GSTs and CCEs, the significance of UGTs has been overlooked because they are generally considered to be a secondary mechanism of enzymatic detoxification [35]. The validation result of the expression of *UGT16*, *UGT22*, *UGT58p* and *UGT65* by qRT-PCR was consistent with that of the transcriptome sequencing (Figure 9b). Among them, *UGT22* was significantly upregulated by 5.29-fold. Therefore, we assumed that these four UGT genes were related to the bifenthrin resistance in *T. urticae*.

The cytochrome P450 monooxygenases participate in the resistance to pyrethroids and organochlorines by catalyzing the oxidation of toxic substances in mites and insects [13,36,37,38]. Previous studies reported that the P450 genes involved in insecticides were mainly in *CYP2*, *CYP4*, *CYP6*, *CYP9* and *CYP12* families [39,40,41]. Yang et al. [41] reported that the up- and down-regulation of P450 genes may function in pesticide metabolism and participate in the homeostatic response of organisms induced by changes in the cellular environment. In this study, *CYP392A11* and *CYP392D2* were upregulated while *CYP392A2p*, *CYP392D7* and *CYP385C4v2* were downregulated in the R_bft strain. Moreover, three P450 genes were identified by qRT-PCR, of which *CYP392A11* was obviously upregulated (Figure 9b). These findings suggest that the upregulated *CYP392A11* may be highly pertinent to bifenthrin resistance. It is noteworthy that *CYP392A11* has been shown to hydroxylate cyenopyrafen and fenpyroximate [42]. Liu et al. [23] reported that *CYP392A1* may participate in the cyflumetofen resistance of *T. cinnabarinus*. However, *CYP392A1* was not significantly up-regulated in the R_cfm strain of *T. urticae* in this study (Figure 9a).

The GSTs of *T. urticae* were classified into six families: Delta, Omega, Zeta, Mu, Kappa and incomplete. Among these families, the GST genes in the Delta family were related to the insect resistance [43]. A prior study found that the GST gene *TuGSTd05* could directly metabolize cyflumetofen and the de-esterified form (AB-1) of cyflumetofen in the highly resistant strain of *T. urticae* [12]. However, in this study, GST genes associated with cyflumetofen resistance were not detected in the R_cfm strain. However, the significantly upregulated *TuGSTd15* and the increased GST activity in the bifenthrin resistant strain may contribute to the resistance of *T. urticae* to bifenthrin (Figure 9b).

**Figure 10:**
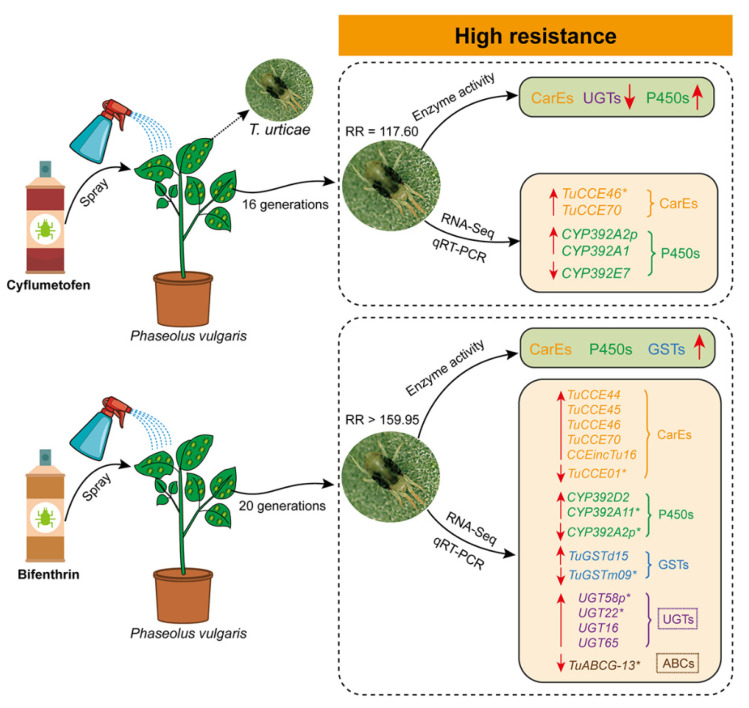
The integrated diagram illustrating the changes of detoxifying enzymes and involved genes in the cyflumetofen- and bifenthrin-resistant *T. urticae* strains based on this study. The up and down arrows indicate the increased/up-regulated and decreased/down-regulated of the parameters, respectively.

In conclusion, in the cyflumetofen-resistant strain, the enzyme activity of UGTs and CarEs decreased, while that of P450s increased. Additionally, the GSTs, CarEs and P450s increased in the bifenthrin-resistant strain. Four upregulated genes (two CCEs and two P450s) and one downregulated P450 gene were involved in the metabolic resistance of the cyflumetofen-resistant strain. However, in the bifenthrin-resistant strain, there were twelve upregulated genes (five CCEs, four UGTs, two P450s and one GST) and four downregulated genes (belonging to CCEs, P450s, GSTs and ABCs) related to its metabolic resistance (Figure 10).

## 4. Materials and Methods

### 4.1. Mites

The original parent strain of *T. urticae* (Lab_SS), which has been reared in the laboratory for more than 6 years without exposure to any acaricide, obtained from the Institute of Plant Protection, Chinese Academy of Agricultural Sciences. The mite was lab-reared on kidney beans (*Phaseolus vulgaris*) in a growth chamber at 20–30 °C, 60–70% RH and L12:D12 h [44]. Then, the colony was maintained at 16:8 (L:D) h at 25 ± 1 °C with 50–60% RH.

### 4.2. Reagents

Acaricides (96% cyflumetofen, CAS: 400882-07-7; and 97.2% bifenthrin CAS: 82657-04-3) were purchased from Sigma-Aldrich Co. (Saint Louis, USA). ELISA kits were purchased from Jiangsu Boshen Biotechnology Co., Ltd. (Nanjing, Jiangsu, China). The Total RNA Kit II was purchased from Omega (Seoul, Korea).

### 4.3. Resistance Selection

Based on the previous method [45], the cyflumetofen or bifenthrin resistance population was selected under laboratory conditions. Cyflumetofen or bifenthrin was diluted with and 40% acetone and water (*v*/*v*). Then, cyflumetofen or bifenthrin was sprayed on cowpea leaves in order to exert a selection pressure. The concentration of each spraying was the last LC_50_. Then, 48 h after spraying, the survivors were transferred to fresh young cowpea leaves. The next selection cycle continued when the population recovered and then increased. After each selection, the concentrations of cyflumetofen or bifenthrin increased accordingly. The spray of cyflumetofen or bifenthrin was conducted at an interval of 14 days.

### 4.4. Bioassays of Cyflumetofen- and Bifenthrin-Resistant Strains of T. urticae

Owing to the phenomenon of the mites escaping into the surrounding water after cyflumetofen treatment, the slide dip method was used to measure the mortality of the cyflumetofen-resistant strain [46]. Specifically, a 2 × 2 cm piece of double-sided adhesive tape was attached to one side of glass microscope slides, and then, 30 adult female mites were affixed to the tape by their dorsal surface. Note that the feet and mouthparts of mites should not be stuck to the tape. The glass microscope slides were placed in a tray covered with wet cotton and then incubated at 26 °C. After 4 h, the dead or inactive individuals were removed and new active female adults were supplemented. Then, five concentrations of cyflumetofen obtained by diluting cyflumetofen solution with water and 40% acetone and the control (i.e., water and 40% acetone) were used for testing. Each concentration had three replicates. Slides were dipped into the solution of cyflumetofen for 5 s. Then the slides were taken out and placed on a paper towel to dry. The extra solution was moved from the slides with filter paper. The treated slides, lined with slightly moistened paper towels, were left in the plastic tray with a cover and incubated under the condition of 16:8 (L:D) h at 25 ± 1 °C with 50–60% RH. The mortality criterion used for this method was the inability to move a leg when lightly prodded. After 24 h, the mortality of the cyflumetofen-resistant strain was determined using a microscope. 

The mortality of the bifenthrin-resistant strain was measured according to the leaf residual toxicity method recommended by the FAO [46]. Briefly, five concentrations (with a 2-fold increase between concentrations) of bifenthrin were obtained by diluting the stock solution of bifenthrin (dissolved in acetone) with 0.1% Tween water and 40% acetone. The control contained 0.1% Tween water and 40% acetone. Then, cowpea leaf discs (4 cm in diameter) were dipped into the solution of bifenthrin for 10 s and subsequently placed on Petri dishes filled with water to dry naturally. Furthermore, 20 adult females of *T. urticae* were gently transported to each of the leaves using a soft brush. The petri dishes were kept in incubators under a cycle of 16:8 (L:D) h at 25 ± 1 °C and 50–60% RH. After 48 h, the mortality of the cyflumetofen-resistant strain was determined using a microscope. The mortality criterion was the same as the abovementioned. 

The LC_50_ value was determined by the corrected mortality calculated with Abbott’s formula. The bifenthrin-resistant strain (R_bft) and the cyflumetofen-resistant strain (R_cfm) were cultivated following the abovementioned process. The resistance ratio (RR) was calculated as the following [47]:

RR = LC_50_ of resistant population/LC_50_ of susceptible population

### 4.5. Determination of the Activity of the Detoxification Enzymes UGTs, GSTs, CarEs and P450s

The activity of UDP-glucuronosyltransferase (UGTs), glutathione-S-transferases (GSTs), carboxylesterase (CarEs) and cytochrome P450 monooxygenases (P450s) was assayed with ELISA kits [48,49,50]. In detail, about 250 female adults (2.5–3.3 mg) were rinsed with cold phosphate buffer (PBS) (pH = 7.4), then dried with absorbent paper and finally put into a 5-milliliter homogenate tube. Then, the homogenization medium was added into the homogenate tube with a ratio of weight (mg, i.e., mites) to volume (mL, i.e., homogenization medium) of 1:9 and evenly grinded. Subsequently, the obtained homogenate was centrifuged at 3000 r/min for 10–15 min, and the supernatant was used for further analysis. The Bicinchoninic Acid (BCA) protein content determination kit was used to test the protein content of the supernatant. Then, the Insect UGTs ELISA kit (BS-E19272O2, Jiangsu Boshen Biotechnology Co., Ltd., Nanjing, Jiangsu, China), Insect GSTs ELISA kit (BS-E19119O2, Jiangsu Boshen Biotechnology Co., Ltd., Nanjing, Jiangsu, China), Insect CES ELISA kit (BS-E19073O2, Jiangsu Boshen Biotechnology Co., Ltd., Nanjing, Jiangsu, China) and InsectCYP450Oase ELISA kit (BS-E19093O2, Jiangsu Boshen Biotechnology Co., Ltd., Nanjing, Jiangsu, China) were applied to determine the enzyme activity of UGTs, GSTs, CarEs and P450s, respectively. 

### 4.6. Transcriptome Sequencing

#### 4.6.1. Total RNA Extraction, Library Construction and RNA-Seq

Three biological replicates of 200 female *T. urticae* adults from three strains, namely Lab_SS, R_cfm and R_bft, were used for the total RNA extraction with the Total RNA Kit II (R6830-02, Omega, Seoul, Korea) [51,52]. The quality of total RNA was assessed using 1% agarose gel electrophoresis. Then, the concentration and purity of the nine extracted RNA samples were tested using a NanoDrop One device (Thermo Scientific, USA). First, the Standard Sensitivity RNA Analysis Kit (15 nt) was used to detect the concentration of total RNA and 28S/18S and RIN values to determine whether the samples met the requirements for database construction. Then, total RNA was processed with rRNA depletion to obtain purified RNA. Subsequently, the enriched mRNA was fragmented to synthesize first-strand cDNA and the second cDNA, and the modified double-stranded DNA was enriched by PCR amplification. The PCR product was thermally denaturized into single-strand cDNA, and then, the single-strand DNA was cycled with a bridge primer to obtain a single-strand circular cDNA library. Finally, the constructed cDNA libraries were sequenced in the DNBSEQ platform of BGI.

#### 4.6.2. Transcriptome Sequencing Data Analysis

During the quality control of sequencing, clean reads were obtained by filtering the raw reads of low quality, contaminated connectors, and high N content of unknown bases. Then, the clean reads were compared against the reference genome sequence (https://bioinformatics.psb.ugent.be/orcae/overview/Tetur accessed on 10 November 2022) using HISAT [53] and were mapped to the reference gene sequence using Bowtie2 [54]. Subsequently, the expression levels of genes and transcripts were calculated with RSEM [55]. The Pearson correlation between each set of two samples was calculated with the cor function in R. Additionally, a principal component analysis (PCA) was performed using the princomp function, and all genes were clustered with the hclust function. The differential genes in this study were detected by the DEseq2 method based on negative binomial distribution. Differentially expressed genes (DEGs) were determined between susceptible and resistant mite populations under the fold change (FC) ≥ 2 and false discovery rate (FDR) < 0.05. 

#### 4.6.3. Transcriptome Sequencing Data Analysis

According to the results of the pairwise comparison of differentially expressed genes, a GO analysis was implemented by molecular function, cellular component and biological processing [21,22]. In addition, functional metabolic pathways were identified using the KEGG pathway database [21].

### 4.7. Quantitative Real-Time PCR (qRT-PCR) Analysis

To verify the detoxification enzyme gene results from DNBSEQ sequencing of BGI, five genes of the cyflumetofen-resistant strain and 16 genes of the bifenthrin-resistant strain were randomly selected. The gene-specific primers designed for the target genes are listed in Table 4. Cyclophilin A (tetur01g12670) [56], the most stable housekeeping gene, was used as the reference gene for the qRT-PCR analysis. In each strain, the total RNA was extracted from approximately 200 female *T. urticae* adults using the Total RNA Kit II (R6830-02, Omega, Seoul, Korea) following the manufacturer’s instructions. The extracted RNA was quantified and then used for qRT-PCR. The cDNA was synthesized using the Reverse Transcriptase M-MLV (Omega). The cDNA was synthesized using M-MLV Reverse Transcriptase. The reverse transcriptional system consisted of 10 μL of total RNA, 4 μL of PrimeScript RT Master Mix forward primer (5×) and 6 μL of RNase-free ddH_2_O. The qRT-PCR was performed in a 25-microliter qPCR reaction system consisting of 12.5 μL SYBR Premix Ex TaqIII, 8.5 μL ddH_2_O, 1 μL of each primer (forward and reverse) and 2 μL synthesized cDNA. The condition of the reaction started with 95 °C for 30 s, followed by 40 cycles with 95 °C for 5 s and 60 °C for 30 s. Melting curves were obtained by increasing the temperature from 60 to 95 °C (0.5 °C/s). There were three biological and technique replicates in the qPCR analysis. The relative gene expression levels were calculated by the 2^-ΔΔCt^ method [57].

### 4.8. Statistical Analysis

One-way analysis of variance (ANOVA) (Tukey for data with homogeneous variances or Games–Howell multi-comparisons for data with heterogeneous variances) was conducted to compare data differences at a significance threshold of *p* < 0.05 in SPSS (Version 25.0, IBM Corp., New York, NY, USA). The mean ± standard error (n = 3) was shown in the figures. The mortality rates of the mites were analyzed using SPSS with the probit analysis. Then, the estimation of the median lethal concentration (LC_50_) and its 95% confidence limits were obtained.

## Figures and Tables

**Table 1:** Selection of the cyflumetofen- and bifenthrin-resistant strains of *T. urticae.*

Acaricide	Generation	Regression	LC_50_ (mg/L) (95% CI)	Resistance Ratio
Cyflumetofen	F_0_	y = 4.14 + 1.10x	6.02 (4.29~8.43)	-
	F_16_	y = −6.65 + 4.09x	707.95 (646.64~775.09)	117.60
Bifenthrin	F_0_	y = −0.21 + 2.46x	125.04 (103.19~151.52)	-
	F_20_	-	>20000	>159.95

**Table 2:** Summary of the RNA sequencing and assembly of *T. urticae.*

Samples	Total Raw Reads (Mb)	Total Clean Reads (Mb)	Clean Reads Ratio (%)	Mapping Rate (%)	Clean Reads Q20 (%)	Clean Reads Q30 (%)
Lab_SS1	45.57	42.24	92.68	63.45	94.94	88.86
Lab_SS2	47.33	42.83	90.50	62.02	94.55	88.10
Lab_SS3	45.57	42.26	92.72	64.88	94.67	88.30
R_cfm1	47.33	43.11	91.10	67.04	94.72	88.42
R_cfm2	42.65	38.51	90.30	70.27	94.80	88.53
R_cfm3	47.33	43.10	91.07	66.80	94.84	88.66
R_bft1	49.08	42.81	87.22	49.29	95.17	89.34
R_bft2	47.33	43.10	91.08	54.89	94.85	88.69
R_bft3	45.57	42.22	92.65	69.80	94.70	88.36

**Table 3:** The DEGs of detoxification enzymes in the cyflumetofen-resistant strain and the bifenthrin-resistant strain of *T. urticae.*

	Detoxification Enzymes	Gene ID	log_2_ (R_cfm or R_bft /Lab_SS)	Symbol
Lab_SS vs R_cfm	CCEs	tetur17g00350	1.77	TuCCE46
		tetur207g00010	1.28	TuCCE70
	P450s	tetur07g06440	1.64	CYP392A2p
		tetur07g06410	1.10	CYP392A1
		tetur27g00340	−1.32	CYP392E7
Lab_SS vs R_bft	CCEs	tetur17g00350	3.68	TuCCE46
		tetur207g00010	3.09	TuCCE70
		tetur207g00020	2.65	CCEincTu16
		tetur17g00300	2.63	TuCCE45
		tetur17g00080	2.21	TuCCE44
		tetur17g00360	2.04	CCEincTu08
		tetur35g00180	1.89	CCEincTu13
		tetur35g00200	1.77	TuCCE65
		tetur04g06770	1.53	TuCCE25
		tetur01g14180	1.26	TuCCE12
		tetur11g05770	1.15	TuCCE35
		tetur16g02380	−1.01	TuCCE40
		tetur04g02550	−1.04	TuCCE22
		tetur30g01290	−1.26	TuCCE61
		tetur01g08680	−1.46	TuCCE01
	UGTs	tetur04g07630	3.31	UGT16
		tetur05g00080	2.83	UGT22
		tetur21g01400	2.29	UGT58p
		tetur22g00420	1.75	UGT65
		tetur08g00190	1.73	UGT40
		tetur09g01660	1.69	UGT
		tetur05g09325	1.56	UGT
		tetur05g00090	1.30	UGT23
		tetur22g00510	1.17	UGT69
	P450s	tetur03g04990	2.04	CYP392D2
		tetur03g00970	1.75	CYP392A11
		tetur03g09961	−1.14	CYP392D7
		tetur46g00150	−1.44	CYP385C4v2
		tetur07g06440	−1.89	CYP392A2p
	GSTs	tetur31g01330	1.88	TuGSTd15
		tetur05g05260	1.65	TuGSTm09
		tetur26g01510	1.49	TuGSTd13
		tetur05g05250	1.48	TuGSTm08
	ABCs	tetur09g01970	1.35	TuABCG-13
		tetur18g00230	1.14	TuABCH-13
		tetur11g02120	1.06	TuABCC-28

**Table 4:** qPCR primers of the selected genes.

Gene	Forward Primer	Reverse Primer
tetur03g04990	TCTAAAGGACCAAGAGGAG	ACGATGGGTTTGATAATGT
tetur04g07630	ATCAGGACCTCCACCATTT	AATCTATTCGGCACCAACC
tetur05g00080	AACATCGCTTCATCGTCTC	CAATCAATCTTGCCACTTC
tetur21g01400	GGACCAAGAGGAGACGAAC	TTCAGCATACGATGGGTTT
tetur17g00350	TTCCTTATGCGAAGCCAACG	AACGGACCAAGATCCAGCA
tetur207g00010	AAAAGGACCAGCGAAGACT	AACGGACCAAGATCCAGCA
tetur207g00020	TATGTTTTCGGTTTACCTC	TTTCCCCAATCATCTATG
tetur17g00300	CAAGAAGAACCAGCGAAGA	CTGACCAAGATCCAGCAGA
tetur17g00080	AACATCGCTTCATCGTCTC	CAATCAATCTTGCCACTTC
tetur17g00360	TCAAACAAACTACGACCAG	CTCCGAAGTAGATGAAACC
tetur01g08680	TTTTGATAATTCGGTTGACG	ACGATATTGGCTTAGGAGG
tetur22g00420	ATGGTCCACCTCTTCGTTC	TTCCCATTGAGACATAGATAAGC
tetur03g00970	AGGAAGCAATTCGCTGATA	GCCAACAATAGGAAGACCC
tetur07g06440	CTCGTCCAATATCCTCAAT	AGCACTAAATGGCACTAAA
tetur31g01330	GATTGGTTTACGCTGTCCT	GTTTCGTTTCGATAAGATTG
tetur05g05260	AGGTGCTGTTGAGCCTATT	TTTCTTGGTCCTAAATCGT
tetur09g01970	ATGTTGCTGGCTGAGGGTC	ATGGCTTCTTGATTGTAGTCCTG
tetur27g00340	GGCTTAGGAAAGAGTGAAA	CGAAATACGGAAACAGACC
tetur01g12670	CTTCAAGGCGGTGACTTTACC	CCATTGAAAGGATACCTGGTCC

## Data Availability

Any data or material that support the findings of this study can be made available by the corresponding author upon request.

## Supplementary Materials

The following supporting information can be downloaded at: https://www.mdpi.com/article/10.3390/ijms232416220/s1.
