# Supplementary material for: Molecular Mechanisms Underlying Metabolic Resistance to Cyflumetofen and Bifenthrin in Tetranychus urticae Koch on Cowpea
Source: Int J Mol Sci. 2022 Dec 19;23(24):16220. doi: 10.3390/ijms232416220 (PMC9787285; doi:10.3390/ijms232416220)
Supplement: Supplementary file 1 [file ijms-23-16220-s001.zip › ijms-1990449-supplementary.pdf]

Table S1. Summary of the GO enrichment analysis of the differentially expressed genes in the cyflumetofen-resistant strains

	GO ontology term	Cluster frequency	Genome frequency of use	Correct P-value
**Biological process**		out of 26 genes	out of 4351 genes	
	DNA integration	5 (19.23%)	28 (0.64%)	0.000213
	10-formyltetrahydrofolate metabolic process	2 (7.69%)	2 (0.05%)	0.002009
	10-formyltetrahydrofolate catabolic process	2 (7.69%)	2 (0.05%)	0.002009
	folic acid-containing compound catabolic process	2 (7.69%)	2 (0.05%)	0.002009
	pteridine-containing compound catabolic process	2 (7.69%)	2 (0.05%)	0.002009
	dicarboxylic acid catabolic process	2 (7.69%)	2 (0.05%)	0.002009
	cellular modified amino acid catabolic process	2 (7.69%)	4 (0.09%)	0.008702
	DNA metabolic process	6 (23.08%)	186 (4.27%)	0.019343
	folic acid-containing compound metabolic process	2 (7.69%)	8 (0.18%)	0.023168
	tetrahydrofolate metabolic process	2 (7.69%)	8 (0.18%)	0.023168
	autophagy	3 (11.54%)	37 (0.85%)	0.028674
	process utilizing autophagic mechanism	3 (11.54%)	37 (0.85%)	0.028674
	one-carbon metabolic process	2 (7.69%)	11 (0.25%)	0.035657
	pteridine-containing compound metabolic process	2 (7.69%)	12 (0.28%)	0.036516
	dicarboxylic acid metabolic process	2 (7.69%)	13 (0.30%)	0.041514
**Cellular component**		out of 38 genes	out of 5997 genes	
	nucleosome	3 (7.89%)	24 (0.40%)	0.016905
	DNA packaging complex	3 (7.89%)	26 (0.43%)	0.019343
	protein-DNA complex	3 (7.89%)	31 (0.52%)	0.023168
**Molecular function**		out of 65 genes	out of 7398 genes	
	aspartic-type endopeptidase activity	5 (7.69%)	37 (0.50%)	0.002009
	aspartic-type peptidase activity	5 (7.69%)	37 (0.50%)	0.002009
	pigment binding	5 (7.69%)	45 (0.61%)	0.00217

formyltetrahydrofolate dehydrogenase activity	2 (3.08%)	2 (0.03%)	0.003558
endopeptidase activity	10 (15.38%)	333 (4.50%)	0.019343
peptidase activity	13 (20.00%)	537 (7.26%)	0.019343
small conductance calcium-activated potassium channel activity	2 (3.08%)	7 (0.09%)	0.033008
calcium activated cation channel activity	2 (3.08%)	8 (0.11%)	0.035657
calcium-activated potassium channel activity	2 (3.08%)	8 (0.11%)	0.035657
hydroxymethyl-, formyl- and related transferase activity	2 (3.08%)	8 (0.11%)	0.035657
ion gated channel activity	2 (3.08%)	8 (0.11%)	0.035657
peptidase activity, acting on L-amino acid peptides	11 (16.92%)	484 (6.54%)	0.046555
cysteine-type endopeptidase inhibitor activity	3 (4.62%)	34 (0.46%)	0.048154

Table S2. Summary of the GO enrichment analysis of the differentially expressed genes in the bifenthrin-resistant strains

	GO ontology term	Cluster frequency	Genome frequency of use	Correct P-value
**Biological process**		out of 105 genes	out of 4351 genes	
	sphingolipid metabolic process	11 (10.48%)	73 (1.68%)	0.00049
	membrane lipid metabolic process	12 (11.43%)	99 (2.28%)	0.001115
	dicarboxylic acid metabolic process	5 (4.76%)	13 (0.30%)	0.002063
	cellular lipid metabolic process	17 (16.19%)	267 (6.14%)	0.022646
	ganglioside metabolic process	4 (3.81%)	16 (0.37%)	0.025211
	lipid metabolic process	21 (20.00%)	398 (9.15%)	0.025211
	glycosphingolipid metabolic process	4 (3.81%)	16 (0.37%)	0.025211
	ganglioside catabolic process	4 (3.81%)	16 (0.37%)	0.025211
	10-formyltetrahydrofolate metabolic process	2 (1.90%)	2 (0.05%)	0.025211
	10-formyltetrahydrofolate catabolic process	2 (1.90%)	2 (0.05%)	0.025211
	folic acid-containing compound catabolic process	2 (1.90%)	2 (0.05%)	0.025211
	glycolipid catabolic process	4 (3.81%)	16 (0.37%)	0.025211
	pteridine-containing compound catabolic process	2 (1.90%)	2 (0.05%)	0.025211
	dicarboxylic acid catabolic process	2 (1.90%)	2 (0.05%)	0.025211
	glycosphingolipid catabolic process	4 (3.81%)	16 (0.37%)	0.025211
	regulation of catalytic activity	9 (8.57%)	91 (2.09%)	0.025211
	folic acid-containing compound metabolic process	3 (2.86%)	8 (0.18%)	0.026068
	tetrahydrofolate metabolic process	3 (2.86%)	8 (0.18%)	0.026068
	regulation of molecular function	9 (8.57%)	102 (2.34%)	0.026068
	ceramide catabolic process	4 (3.81%)	18 (0.41%)	0.027103
	ceramide metabolic process	4 (3.81%)	21 (0.48%)	0.042539
**Cellular component**		out of 138 genes	out of 5997 genes	
	integral component of membrane	100 (72.46%)	3577 (59.65%)	0.035415
	intrinsic component of membrane	100 (72.46%)	3579 (59.68%)	0.035415
**Molecular**		out of 245 genes	out of 7398 genes	

**function**	pigment binding	11 (4.49%)	45 (0.61%)	0.000158
	catalytic activity	165 (67.35%)	4153 (56.14%)	0.022646
	peptidase activity	34 (13.88%)	537 (7.26%)	0.022646
	hydrolase activity	84 (34.29%)	1779 (24.05%)	0.022646
	cysteine-type endopeptidase activity	8 (3.27%)	51 (0.69%)	0.025211
	glucosylceramidase activity	7 (2.86%)	41 (0.55%)	0.025211
	cysteine-type peptidase activity	14 (5.71%)	143 (1.93%)	0.025211
	endopeptidase activity	23 (9.39%)	333 (4.50%)	0.026068
	formyltetrahydrofolate dehydrogenase activity	2 (0.82%)	2 (0.03%)	0.035415
	peptidase activity, acting on L-amino acid peptides	29 (11.84%)	484 (6.54%)	0.040118

Table S3. Summary of the GO enrichment analysis of the common differentially expressed genes between the Lab_SS-vs-R_cfm group and the Lab_SS-vs-R_bft group

	GO ontology term	Cluster frequency	Genome frequency of use	Correct P-value
		out of 13 genes	out of 4351 genes	
Biological process	10-formyltetrahydrofolate metabolic process	2 (15.38%)	2 (0.05%)	0.000419
	10-formyltetrahydrofolate catabolic process	2 (15.38%)	2 (0.05%)	0.000419
	folic acid-containing compound catabolic process	2 (15.38%)	2 (0.05%)	0.000419
	pteridine-containing compound catabolic process	2 (15.38%)	2 (0.05%)	0.000419
	dicarboxylic acid catabolic process	2 (15.38%)	2 (0.05%)	0.000419
	cellular modified amino acid catabolic process	2 (15.38%)	4 (0.09%)	0.001879
	folic acid-containing compound metabolic process	2 (15.38%)	8 (0.18%)	0.006968
	tetrahydrofolate metabolic process	2 (15.38%)	8 (0.18%)	0.006968
	one-carbon metabolic process	2 (15.38%)	11 (0.25%)	0.012380
	pteridine-containing compound metabolic process	2 (15.38%)	12 (0.28%)	0.013595
	dicarboxylic acid metabolic process	2 (15.38%)	13 (0.30%)	0.014806
	nucleosome assembly	2 (15.38%)	16 (0.37%)	0.019642
	chromatin assembly	2 (15.38%)	17 (0.39%)	0.020834
	immune system process	2 (15.38%)	20 (0.46%)	0.024387
	DNA packaging	2 (15.38%)	20 (0.46%)	0.024387
	chromatin assembly or disassembly	2 (15.38%)	20 (0.46%)	0.024387
	cellular modified amino acid metabolic process	2 (15.38%)	22 (0.51%)	0.026735
	nucleosome organization	2 (15.38%)	22 (0.51%)	0.026735
	protein-DNA complex assembly	2 (15.38%)	28 (0.64%)	0.036379
	regulation of immune effector process	1 (7.69%)	2 (0.05%)	0.036401
	inflammatory response	1 (7.69%)	2 (0.05%)	0.036401
	post-embryonic hemopoiesis	1 (7.69%)	2 (0.05%)	0.036401
	larval lymph gland hemopoiesis	1 (7.69%)	2 (0.05%)	0.036401
	regulation of CD4-positive, alpha-beta T cell differentiation	1 (7.69%)	2 (0.05%)	0.036401

negative regulation of CD4-positive, alpha-beta T cell differentiation	1 (7.69%)	2 (0.05%)	0.036401
regulation of T cell differentiation	1 (7.69%)	2 (0.05%)	0.036401
negative regulation of T cell differentiation	1 (7.69%)	2 (0.05%)	0.036401
regulation of lymphocyte differentiation	1 (7.69%)	2 (0.05%)	0.036401
negative regulation of lymphocyte differentiation	1 (7.69%)	2 (0.05%)	0.036401
regulation of alpha-beta T cell activation	1 (7.69%)	2 (0.05%)	0.036401
negative regulation of alpha-beta T cell activation	1 (7.69%)	2 (0.05%)	0.036401
regulation of alpha-beta T cell differentiation	1 (7.69%)	2 (0.05%)	0.036401
negative regulation of alpha-beta T cell differentiation	1 (7.69%)	2 (0.05%)	0.036401
lymph gland development	1 (7.69%)	2 (0.05%)	0.036401
negative regulation of immune response	1 (7.69%)	2 (0.05%)	0.036401
DNA conformation change	2 (15.38%)	29 (0.67%)	0.036401
protein-DNA complex subunit organization	2 (15.38%)	34 (0.78%)	0.036401
regulation of leukocyte differentiation	1 (7.69%)	2 (0.05%)	0.036401
negative regulation of leukocyte differentiation	1 (7.69%)	2 (0.05%)	0.036401
negative regulation of hemopoiesis	1 (7.69%)	2 (0.05%)	0.036401
regulation of CD4-positive, alpha-beta T cell activation	1 (7.69%)	2 (0.05%)	0.036401
negative regulation of CD4-positive, alpha-beta T cell activation	1 (7.69%)	2 (0.05%)	0.036401
negative regulation of immune system process	1 (7.69%)	3 (0.07%)	0.041308
regulation of leukocyte activation	1 (7.69%)	3 (0.07%)	0.041308
negative regulation of leukocyte activation	1 (7.69%)	3 (0.07%)	0.041308
organic acid catabolic process	2 (15.38%)	46 (1.06%)	0.041308
regulation of cell-cell adhesion	1 (7.69%)	3 (0.07%)	0.041308
negative regulation of cell-cell adhesion	1 (7.69%)	3 (0.07%)	0.041308
carboxylic acid catabolic process	2 (15.38%)	46 (1.06%)	0.041308

	regulation of T cell activation	1 (7.69%)	3 (0.07%)	0.041308
	regulation of cell activation	1 (7.69%)	3 (0.07%)	0.041308
	negative regulation of cell activation	1 (7.69%)	3 (0.07%)	0.041308
	negative regulation of T cell activation	1 (7.69%)	3 (0.07%)	0.041308
	regulation of lymphocyte activation	1 (7.69%)	3 (0.07%)	0.041308
	negative regulation of lymphocyte activation	1 (7.69%)	3 (0.07%)	0.041308
	regulation of leukocyte cell-cell adhesion	1 (7.69%)	3 (0.07%)	0.041308
	negative regulation of leukocyte cell-cell adhesion	1 (7.69%)	3 (0.07%)	0.041308
	regulation of hemopoiesis	1 (7.69%)	3 (0.07%)	0.041308
Cellular component		out of 18 genes	out of 5997 genes	
	nucleosome	2 (11.11%)	24 (0.40%)	0.031312
	DNA packaging complex	2 (11.11%)	26 (0.43%)	0.033679
	protein-DNA complex	2 (11.11%)	31 (0.52%)	0.036401
Molecular function		out of 38 genes	out of 7398 genes	
	pigment binding	5 (13.16%)	45 (0.61%)	0.000419
	formyltetrahydrofolate dehydrogenase activity	2 (5.26%)	2 (0.03%)	0.001119
	hydroxymethyl-, formyl- and related transferase activity	2 (5.26%)	8 (0.11%)	0.015370
	oxidoreductase activity, acting on the CH-NH group of donors, NAD or NADP as acceptor	2 (5.26%)	15 (0.20%)	0.033679
	oxidoreductase activity, acting on the CH-NH group of donors	2 (5.26%)	18 (0.24%)	0.036401
	oxidoreductase activity, acting on the aldehyde or oxo group of donors, NAD or NADP as acceptor	2 (5.26%)	30 (0.41%)	0.046470

Table S4. Summary of the KEGG enrichment analysis of the differentially expressed genes in cyflumetofen-resistant strains

Pathway	DEGs with pathway annotation (64)	All genes with pathway annotation (6770)	P-value	Q-value	Pathway ID
Steroid biosynthesis	4 (6.25%)	62 (0.92%)	0.002688	0.117587	ko00100
Lysosome	11 (17.19%)	464 (6.85%)	0.003755	0.117587	ko04142
Salivary secretion	5 (7.81%)	120 (1.77%)	0.005332	0.117587	ko04970
Notch signaling pathway	4 (6.25%)	75 (1.11%)	0.005345	0.117587	ko04330
Autophagy-other	3 (4.69%)	46 (0.68%)	0.009159	0.161202	ko04136
One carbon pool by folate	2 (3.13%)	22 (0.32%)	0.017995	0.232801	ko00670
Lysine degradation	5 (7.81%)	163 (2.41%)	0.018518	0.232801	ko00310
mRNA surveillance pathway	4 (6.25%)	120 (1.77%)	0.026382	0.286526	ko03015
Antigen processing and presentation	4 (6.25%)	124 (1.83%)	0.029304	0.286526	ko04612
Autophagy-animal	5 (7.81%)	197 (2.91%)	0.037868	0.333242	ko04140
Fc gamma R-mediated phagocytosis	3 (4.69%)	89 (1.31%)	0.051538	0.412301	ko04666
Linoleic acid metabolism	3 (4.69%)	100 (1.48%)	0.068282	0.413588	ko00591
Folate biosynthesis	2 (3.13%)	47 (0.69%)	0.072494	0.413588	ko00790
Regulation of actin cytoskeleton	4 (6.25%)	169 (2.50%)	0.075115	0.413588	ko04810
Cholesterol metabolism	4 (6.25%)	169 (2.50%)	0.075115	0.413588	ko04979
Fructose and mannose metabolism	2 (3.13%)	48 (0.71%)	0.075198	0.413588	ko00051
Galactose metabolism	2 (3.13%)	50 (0.74%)	0.080702	0.417749	ko00052
Ovarian steroidogenesis	3 (4.69%)	112 (1.65%)	0.089006	0.435143	ko04913
Arachidonic acid metabolism	3 (4.69%)	119 (1.76%)	0.102183	0.449606	ko00590
Axon guidance	3 (4.69%)	119 (1.76%)	0.102183	0.449606	ko04360
Toll and Imd signaling pathway	2 (3.13%)	70 (1.03%)	0.141444	0.592718	ko04624
Serotonergic synapse	3 (4.69%)	151 (2.23%)	0.170882	0.626611	ko04726
Other glycan degradation	2 (3.13%)	79 (1.17%)	0.17125	0.626611	ko00511
Inflammatory mediator regulation of TRP channels	3 (4.69%)	153 (2.26%)	0.175557	0.626611	ko04750
Insulin secretion	2 (3.13%)	81 (1.20%)	0.178014	0.626611	ko04911
Circadian rhythm-fly	1 (1.56%)	26 (0.38%)	0.219186	0.714936	ko04711
Adherens junction	2 (3.13%)	93 (1.37%)	0.219355	0.714936	ko04520

Glycerolipid metabolism	2 (3.13%)	99 (1.46%)	0.240352	0.755392	ko00561
Ribosome biogenesis in eukaryotes	2 (3.13%)	107 (1.58%)	0.268496	0.781892	ko03008
ECM-receptor interaction	2 (3.13%)	118 (1.74%)	0.30718	0.781892	ko04512
Prolactin signaling pathway	1 (1.56%)	39 (0.58%)	0.310293	0.781892	ko04917
RNA polymerase	2 (3.13%)	119 (1.76%)	0.310684	0.781892	ko03020
Signaling pathways regulating pluripotency of stem cells	2 (3.13%)	124 (1.83%)	0.328152	0.781892	ko04550
Pentose phosphate pathway	1 (1.56%)	42 (0.62%)	0.329783	0.781892	ko00030
Hedgehog signaling pathway-fly	1 (1.56%)	42 (0.62%)	0.329783	0.781892	ko04341
Endocrine and other factor-regulated calcium reabsorption	1 (1.56%)	42 (0.62%)	0.329783	0.781892	ko04961
B cell receptor signaling pathway	1 (1.56%)	45 (0.66%)	0.34873	0.781892	ko04662
Glycine, serine and threonine metabolism	1 (1.56%)	46 (0.68%)	0.354928	0.781892	ko00260
cGMP-PKG signaling pathway	2 (3.13%)	134 (1.98%)	0.362727	0.781892	ko04022
Focal adhesion	3 (4.69%)	229 (3.38%)	0.368759	0.781892	ko04510
Pentose and glucuronate interconversions	2 (3.13%)	136 (2.01%)	0.36957	0.781892	ko00040
T cell receptor signaling pathway	1 (1.56%)	49 (0.72%)	0.373176	0.781892	ko04660
ErbB signaling pathway	1 (1.56%)	55 (0.81%)	0.40816	0.827601	ko04012
Apoptosis-multiple species	1 (1.56%)	56 (0.83%)	0.413801	0.827601	ko04215
Retinol metabolism	2 (3.13%)	162 (2.39%)	0.455588	0.840817	ko00830
Apoptosis	2 (3.13%)	163 (2.41%)	0.45877	0.840817	ko04210
Leukocyte transendothelial migration	1 (1.56%)	68 (1.00%)	0.477498	0.840817	ko04670
Aminoacyl-tRNA biosynthesis	1 (1.56%)	69 (1.02%)	0.482488	0.840817	ko00970
Longevity regulating pathway-worm	2 (3.13%)	172 (2.54%)	0.486928	0.840817	ko04212
IL-17 signaling pathway	1 (1.56%)	72 (1.06%)	0.497177	0.840817	ko04657
Longevity regulating pathway	1 (1.56%)	75 (1.11%)	0.511455	0.840817	ko04211
Hedgehog signaling pathway	1 (1.56%)	75 (1.11%)	0.511455	0.840817	ko04340
Thyroid hormone signaling pathway	2 (3.13%)	183 (2.70%)	0.520117	0.840817	ko04919
GABAergic synapse	1 (1.56%)	77 (1.14%)	0.520752	0.840817	ko04727
Ubiquitin mediated proteolysis	2 (3.13%)	187 (2.76%)	0.531837	0.840817	ko04120
Sphingolipid metabolism	1 (1.56%)	83 (1.23%)	0.547608	0.840817	ko00600
Melanogenesis	1 (1.56%)	88 (1.30%)	0.568853	0.840817	ko04916
Spliceosome	2 (3.13%)	201 (2.97%)	0.571342	0.840817	ko03040
Renin secretion	1 (1.56%)	90 (1.33%)	0.577073	0.840817	ko04924

Chemokine signaling pathway	1 (1.56%)	91 (1.34%)	0.581125	0.840817	ko04062
Estrogen signaling pathway	1 (1.56%)	95 (1.40%)	0.596954	0.840817	ko04915
Necroptosis	1 (1.56%)	102 (1.51%)	0.623251	0.840817	ko04217
Neurotrophin signaling pathway	1 (1.56%)	103 (1.52%)	0.626867	0.840817	ko04722
RNA degradation	1 (1.56%)	107 (1.58%)	0.640993	0.840817	ko03018
Dopaminergic synapse	1 (1.56%)	107 (1.58%)	0.640993	0.840817	ko04728
Vascular smooth muscle contraction	1 (1.56%)	112 (1.65%)	0.657912	0.840817	ko04270
Hippo signaling pathway	1 (1.56%)	112 (1.65%)	0.657912	0.840817	ko04390
Oxytocin signaling pathway	1 (1.56%)	118 (1.74%)	0.67718	0.840817	ko04921
PI3K-Akt signaling pathway	2 (3.13%)	245 (3.62%)	0.679785	0.840817	ko04151
NOD-like receptor signaling pathway	1 (1.56%)	120 (1.77%)	0.683363	0.840817	ko04621
Synaptic vesicle cycle	1 (1.56%)	121 (1.79%)	0.68641	0.840817	ko04721
Retrograde endocannabinoid signaling	1 (1.56%)	128 (1.89%)	0.706948	0.840817	ko04723
Wnt signaling pathway	1 (1.56%)	132 (1.95%)	0.718083	0.840817	ko04310
Platelet activation	1 (1.56%)	132 (1.95%)	0.718083	0.840817	ko04611
Cell cycle	1 (1.56%)	133 (1.96%)	0.720801	0.840817	ko04110
Pancreatic secretion	1 (1.56%)	135 (1.99%)	0.72616	0.840817	ko04972
mTOR signaling pathway	1 (1.56%)	144 (2.13%)	0.749049	0.855865	ko04150
Tight junction	1 (1.56%)	148 (2.19%)	0.758608	0.855865	ko04530
Insulin signaling pathway	1 (1.56%)	153 (2.26%)	0.770053	0.85778	ko04910
Rap1 signaling pathway	1 (1.56%)	159 (2.35%)	0.783083	0.859753	ko04015
Neuroactive ligand-receptor interaction	1 (1.56%)	163 (2.41%)	0.791364	0.859753	ko04080
Calcium signaling pathway	1 (1.56%)	168 (2.48%)	0.801278	0.859908	ko04020
Phagosome	1 (1.56%)	176 (2.60%)	0.816184	0.865351	ko04145
MAPK signaling pathway	1 (1.56%)	182 (2.69%)	0.826636	0.865999	ko04010
Protein processing in endoplasmic reticulum	1 (1.56%)	217 (3.21%)	0.87692	0.907871	ko04141
cAMP signaling pathway	1 (1.56%)	228 (3.37%)	0.889525	0.910212	ko04024
Endocytosis	1 (1.56%)	254 (3.75%)	0.914485	0.924997	ko04144

Table S5. Summary of the KEGG enrichment analysis of the differentially expressed genes in bifenthrin-resistant strains

Pathway	DEGs with pathway annotation (224)	All genes with pathway annotation (6770)	P-value	Q-value	Pathway ID
Lysosome	44 (19.64%)	464 (6.85%)	9.42E-11	1.79E-08	ko04142
Antigen processing and presentation	18 (8.04%)	124 (1.83%)	1.04E-07	9.89E-06	ko04612
Metabolism of xenobiotics by cytochrome P450	18 (8.04%)	142 (2.10%)	8.34E-07	5.28E-05	ko00980
Pentose and glucuronate interconversions	17 (7.59%)	136 (2.01%)	2.08E-06	9.86E-05	ko00040
Drug metabolism-other enzymes	21 (9.38%)	228 (3.37%)	1.84E-05	0.000699	ko00983
Retinol metabolism	17 (7.59%)	162 (2.39%)	2.25E-05	0.000711	ko00830
Apoptosis	16 (7.14%)	163 (2.41%)	8.87E-05	0.002408	ko04210
Arachidonic acid metabolism	13 (5.80%)	119 (1.76%)	0.00014	0.003334	ko00590
Autophagy-animal	17 (7.59%)	197 (2.91%)	0.000261	0.005514	ko04140
Ascorbate and aldarate metabolism	11 (4.91%)	100 (1.48%)	0.000431	0.008194	ko00053
Drug metabolism-cytochrome P450	13 (5.80%)	135 (1.99%)	0.000493	0.008511	ko00982
Protein export	8 (3.57%)	59 (0.87%)	0.000655	0.009827	ko03060
Phagosome	15 (6.70%)	176 (2.60%)	0.000685	0.009827	ko04145
Renin secretion	10 (4.46%)	90 (1.33%)	0.000724	0.009827	ko04924
One carbon pool by folate	4 (1.79%)	22 (0.32%)	0.005337	0.065558	ko00670
Steroid hormone biosynthesis	10 (4.46%)	118 (1.74%)	0.005521	0.065558	ko00140
Sphingolipid metabolism	8 (3.57%)	83 (1.23%)	0.005915	0.066105	ko00600
Porphyrin and chlorophyll metabolism	9 (4.02%)	103 (1.52%)	0.006836	0.072157	ko00860
Lysine degradation	12 (5.36%)	163 (2.41%)	0.007609	0.076092	ko00310
Pyruvate metabolism	5 (2.23%)	41 (0.61%)	0.010766	0.102276	ko00620
Protein processing in endoplasmic reticulum	14 (6.25%)	217 (3.21%)	0.012586	0.113876	ko04141
Other glycan degradation	7 (3.13%)	79 (1.17%)	0.015213	0.131385	ko00511
Linoleic acid metabolism	8 (3.57%)	100 (1.48%)	0.017332	0.133859	ko00591
NOD-like receptor signaling pathway	9 (4.02%)	120 (1.77%)	0.017613	0.133859	ko04621
Salivary secretion	9 (4.02%)	120 (1.77%)	0.017613	0.133859	ko04970

Cholesterol metabolism	11 (4.91%)	169 (2.50%)	0.024122	0.169746	ko04979
Citrate cycle (TCA cycle)	4 (1.79%)	37 (0.55%)	0.032806	0.22261	ko00020
Glycerophospholipid metabolism	9 (4.02%)	143 (2.11%)	0.047102	0.308602	ko00564
Proximal tubule bicarbonate reclamation	3 (1.34%)	26 (0.38%)	0.053099	0.327659	ko04964
Steroid biosynthesis	5 (2.23%)	62 (0.92%)	0.05346	0.327659	ko00100
Glycine, serine and threonine metabolism	4 (1.79%)	46 (0.68%)	0.064581	0.383448	ko00260
Fc gamma R-mediated phagocytosis	6 (2.68%)	89 (1.31%)	0.073769	0.424732	ko04666
Glyoxylate and dicarboxylate metabolism	3 (1.34%)	31 (0.46%)	0.081523	0.45557	ko00630
Adherens junction	6 (2.68%)	93 (1.37%)	0.087032	0.47246	ko04520
alpha-Linolenic acid metabolism	2 (0.89%)	17 (0.25%)	0.107087	0.565184	ko00592
Selenocompound metabolism	2 (0.89%)	19 (0.28%)	0.129071	0.662795	ko00450
Glycolysis / Gluconeogenesis	4 (1.79%)	62 (0.92%)	0.148435	0.728343	ko00010
Peroxisome	7 (3.13%)	133 (1.96%)	0.151052	0.728343	ko04146
Valine, leucine and isoleucine biosynthesis	1 (0.45%)	5 (0.07%)	0.154887	0.728343	ko00290
Pancreatic secretion	7 (3.13%)	135 (1.99%)	0.159402	0.728343	ko04972
Pentose phosphate pathway	3 (1.34%)	42 (0.62%)	0.161002	0.728343	ko00030
Cholinergic synapse	6 (2.68%)	113 (1.67%)	0.1708	0.754697	ko04725
Glutathione metabolism	4 (1.79%)	67 (0.99%)	0.180507	0.779463	ko00480
Protein digestion and absorption	7 (3.13%)	142 (2.10%)	0.190209	0.802418	ko04974
Toll and Imd signaling pathway	4 (1.79%)	70 (1.03%)	0.20077	0.802418	ko04624
Axon guidance	6 (2.68%)	119 (1.76%)	0.200859	0.802418	ko04360
Cysteine and methionine metabolism	3 (1.34%)	47 (0.69%)	0.202716	0.802418	ko00270
C-type lectin receptor signaling pathway	4 (1.79%)	72 (1.06%)	0.214641	0.804029	ko04625
T cell receptor signaling pathway	3 (1.34%)	49 (0.72%)	0.220059	0.804029	ko04660
Adipocytokine signaling pathway	3 (1.34%)	49 (0.72%)	0.220059	0.804029	ko04920
Insect hormone biosynthesis	2 (0.89%)	27 (0.40%)	0.224282	0.804029	ko00981
Carbohydrate digestion and absorption	2 (0.89%)	27 (0.40%)	0.224282	0.804029	ko04973
Longevity regulating pathway	4 (1.79%)	75 (1.11%)	0.235922	0.824272	ko04211
Inflammatory mediator regulation of TRP channels	7 (3.13%)	153 (2.26%)	0.242943	0.824272	ko04750
Insulin signaling pathway	7 (3.13%)	153 (2.26%)	0.242943	0.824272	ko04910

Retrograde endocannabinoid signaling	6 (2.68%)	128 (1.89%)	0.24919	0.830634	ko04723
ErbB signaling pathway	3 (1.34%)	55 (0.81%)	0.273598	0.878341	ko04012
Ether lipid metabolism	2 (0.89%)	31 (0.46%)	0.273815	0.878341	ko00565
Cell cycle	6 (2.68%)	133 (1.96%)	0.277371	0.878341	ko04110
Fatty acid biosynthesis	2 (0.89%)	33 (0.49%)	0.298587	0.923748	ko00061
Collecting duct acid secretion	2 (0.89%)	34 (0.50%)	0.310929	0.923748	ko04966
Ovarian steroidogenesis	5 (2.23%)	112 (1.65%)	0.312367	0.923748	ko04913
Glucagon signaling pathway	4 (1.79%)	86 (1.27%)	0.317317	0.923748	ko04922
Regulation of actin cytoskeleton	7 (3.13%)	169 (2.50%)	0.326409	0.923748	ko04810
Histidine metabolism	1 (0.45%)	12 (0.18%)	0.332421	0.923748	ko00340
Sulfur relay system	1 (0.45%)	12 (0.18%)	0.332421	0.923748	ko04122
Starch and sucrose metabolism	2 (0.89%)	36 (0.53%)	0.335466	0.923748	ko00500
Hippo signaling pathway-multiple species	2 (0.89%)	36 (0.53%)	0.335466	0.923748	ko04392
Cellular senescence	5 (2.23%)	119 (1.76%)	0.358529	0.962693	ko04218
Natural killer cell mediated cytotoxicity	2 (0.89%)	38 (0.56%)	0.359743	0.962693	ko04650
Prolactin signaling pathway	2 (0.89%)	39 (0.58%)	0.371762	0.981038	ko04917
Serotonergic synapse	6 (2.68%)	151 (2.23%)	0.383256	0.997157	ko04726
Nitrogen metabolism	1 (0.45%)	15 (0.22%)	0.396636	0.997157	ko00910
Aminoacyl-tRNA biosynthesis	3 (1.34%)	69 (1.02%)	0.40105	0.997157	ko00970
Tryptophan metabolism	2 (0.89%)	42 (0.62%)	0.407246	0.997157	ko00380
Renin-angiotensin system	1 (0.45%)	16 (0.24%)	0.416644	0.997157	ko04614
Nicotinate and nicotinamide metabolism	1 (0.45%)	17 (0.25%)	0.435992	0.997157	ko00760
Necroptosis	4 (1.79%)	102 (1.51%)	0.438098	0.997157	ko04217
B cell receptor signaling pathway	2 (0.89%)	45 (0.66%)	0.441739	0.997157	ko04662
Neurotrophin signaling pathway	4 (1.79%)	103 (1.52%)	0.445516	0.997157	ko04722
Autophagy-other	2 (0.89%)	46 (0.68%)	0.452993	0.997157	ko04136
Hedgehog signaling pathway	3 (1.34%)	75 (1.11%)	0.454177	0.997157	ko04340
cGMP-PKG signaling pathway	5 (2.23%)	134 (1.98%)	0.45743	0.997157	ko04022
Ribosome biogenesis in eukaryotes	4 (1.79%)	107 (1.58%)	0.474872	0.997157	ko03008
Fructose and mannose metabolism	2 (0.89%)	48 (0.71%)	0.475106	0.997157	ko00051
AMPK signaling pathway	5 (2.23%)	137 (2.02%)	0.476812	0.997157	ko04152
Osteoclast differentiation	2 (0.89%)	49 (0.72%)	0.485959	0.997157	ko04380

Carbon metabolism	5 (2.23%)	140 (2.07%)	0.495967	0.997157	ko01200
Galactose metabolism	2 (0.89%)	50 (0.74%)	0.496672	0.997157	ko00052
Aldosterone-regulated sodium reabsorption	1 (0.45%)	21 (0.31%)	0.507199	0.997157	ko04960
Fat digestion and absorption	3 (1.34%)	82 (1.21%)	0.51355	0.997157	ko04975
Toll-like receptor signaling pathway	2 (0.89%)	52 (0.77%)	0.517669	0.997157	ko04620
Sphingolipid signaling pathway	5 (2.23%)	145 (2.14%)	0.527292	0.997157	ko04071
FoxO signaling pathway	3 (1.34%)	85 (1.26%)	0.537938	0.997157	ko04068
Longevity regulating pathway-multiple species	3 (1.34%)	85 (1.26%)	0.537938	0.997157	ko04213
Phototransduction	1 (0.45%)	23 (0.34%)	0.53937	0.997157	ko04744
N-Glycan biosynthesis	2 (0.89%)	57 (0.84%)	0.567565	0.997157	ko00510
Circadian rhythm-fly	1 (0.45%)	26 (0.38%)	0.583749	0.997157	ko04711
Chemokine signaling pathway	3 (1.34%)	91 (1.34%)	0.584557	0.997157	ko04062
Oxidative phosphorylation	4 (1.79%)	124 (1.83%)	0.5918	0.997157	ko00190
Hematopoietic cell lineage	1 (0.45%)	27 (0.40%)	0.597575	0.997157	ko04640
Ras signaling pathway	5 (2.23%)	159 (2.35%)	0.610007	0.997157	ko04014
Glycosylphosphatidylinositol (GPI)-anchor biosynthesis	1 (0.45%)	28 (0.41%)	0.610943	0.997157	ko00563
Estrogen signaling pathway	3 (1.34%)	95 (1.40%)	0.613935	0.997157	ko04915
Gastric acid secretion	2 (0.89%)	64 (0.95%)	0.631006	0.997157	ko04971
Fc epsilon RI signaling pathway	1 (0.45%)	30 (0.44%)	0.636368	0.997157	ko04664
Glycerolipid metabolism	3 (1.34%)	99 (1.46%)	0.641893	0.997157	ko00561
VEGF signaling pathway	1 (0.45%)	31 (0.46%)	0.648453	0.997157	ko04370
Calcium signaling pathway	5 (2.23%)	168 (2.48%)	0.658555	0.997157	ko04020
Vitamin digestion and absorption	4 (1.79%)	135 (1.99%)	0.658778	0.997157	ko04977
PPAR signaling pathway	2 (0.89%)	69 (1.02%)	0.671741	0.997157	ko03320
Mannose type O-glycan biosynthesis	1 (0.45%)	35 (0.52%)	0.692924	0.997157	ko00515
beta-Alanine metabolism	1 (0.45%)	36 (0.53%)	0.703137	0.997157	ko00410
p53 signaling pathway	1 (0.45%)	36 (0.53%)	0.703137	0.997157	ko04115
Th17 cell differentiation	1 (0.45%)	36 (0.53%)	0.703137	0.997157	ko04659
Progesterone-mediated oocyte maturation	2 (0.89%)	74 (1.09%)	0.70877	0.997157	ko04914
Thyroid hormone synthesis	2 (0.89%)	76 (1.12%)	0.72258	0.997157	ko04918
Vascular smooth muscle contraction	3 (1.34%)	112 (1.65%)	0.722758	0.997157	ko04270
Hippo signaling pathway	3 (1.34%)	112 (1.65%)	0.722758	0.997157	ko04390

Biosynthesis of amino acids	2 (0.89%)	77 (1.14%)	0.729277	0.997157	ko01230
GABAergic synapse	2 (0.89%)	77 (1.14%)	0.729277	0.997157	ko04727
HIF-1 signaling pathway	2 (0.89%)	80 (1.18%)	0.748551	0.997157	ko04066
Jak-STAT signaling pathway	1 (0.45%)	41 (0.61%)	0.749347	0.997157	ko04630
Hedgehog signaling pathway-fly	1 (0.45%)	42 (0.62%)	0.757691	0.997157	ko04341
mRNA surveillance pathway	3 (1.34%)	120 (1.77%)	0.765047	0.997157	ko03015
Arginine and proline metabolism	1 (0.45%)	43 (0.64%)	0.765758	0.997157	ko00330
Synaptic vesicle cycle	3 (1.34%)	121 (1.79%)	0.769948	0.997157	ko04721
Cell adhesion molecules (CAMs)	2 (0.89%)	84 (1.24%)	0.772414	0.997157	ko04514
cAMP signaling pathway	6 (2.68%)	228 (3.37%)	0.7726	0.997157	ko04024
Focal adhesion	6 (2.68%)	229 (3.38%)	0.776181	0.997157	ko04510
Rap1 signaling pathway	4 (1.79%)	159 (2.35%)	0.777835	0.997157	ko04015
Phototransduction-fly	1 (0.45%)	45 (0.66%)	0.781099	0.997157	ko04745
RNA transport	7 (3.13%)	265 (3.91%)	0.781234	0.997157	ko03013
ABC transporters	3 (1.34%)	124 (1.83%)	0.784155	0.997157	ko02010
Signaling pathways regulating pluripotency of stem cells	3 (1.34%)	124 (1.83%)	0.784155	0.997157	ko04550
Mineral absorption	1 (0.45%)	46 (0.68%)	0.788391	0.997157	ko04978
Glutamatergic synapse	2 (0.89%)	88 (1.30%)	0.794287	0.997157	ko04724
Melanogenesis	2 (0.89%)	88 (1.30%)	0.794287	0.997157	ko04916
Folate biosynthesis	1 (0.45%)	47 (0.69%)	0.79544	0.997157	ko00790
Bile secretion	2 (0.89%)	91 (1.34%)	0.809456	0.997157	ko04976
Platelet activation	3 (1.34%)	132 (1.95%)	0.818538	0.997157	ko04611
Hippo signaling pathway-fly	2 (0.89%)	93 (1.37%)	0.81901	0.997157	ko04391
Olfactory transduction	1 (0.45%)	51 (0.75%)	0.821376	0.997157	ko04740
PI3K-Akt signaling pathway	6 (2.68%)	245 (3.62%)	0.82787	0.997157	ko04151
Fatty acid metabolism	3 (1.34%)	137 (2.02%)	0.837588	0.997157	ko01212
Phosphatidylinositol signaling system	2 (0.89%)	98 (1.45%)	0.841049	0.997157	ko04070
Fatty acid degradation	1 (0.45%)	55 (0.81%)	0.844036	0.997157	ko00071
Regulation of lipolysis in adipocytes	1 (0.45%)	55 (0.81%)	0.844036	0.997157	ko04923
Apoptosis-multiple species	1 (0.45%)	56 (0.83%)	0.849238	0.997157	ko04215
Th1 and Th2 cell differentiation	1 (0.45%)	56 (0.83%)	0.849238	0.997157	ko04658
TNF signaling pathway	1 (0.45%)	56 (0.83%)	0.849238	0.997157	ko04668
Fatty acid elongation	1 (0.45%)	60 (0.89%)	0.868377	0.997157	ko00062

Oocyte meiosis	2 (0.89%)	106 (1.57%)	0.871309	0.997157	ko04114
Long-term potentiation	1 (0.45%)	61 (0.90%)	0.872771	0.997157	ko04720
Dopaminergic synapse	2 (0.89%)	107 (1.58%)	0.874697	0.997157	ko04728
TGF-beta signaling pathway	1 (0.45%)	63 (0.93%)	0.881126	0.997157	ko04350
Complement and coagulation cascades	1 (0.45%)	63 (0.93%)	0.881126	0.997157	ko04610
Apoptosis-fly	1 (0.45%)	65 (0.96%)	0.888934	0.997157	ko04214
Ribosome	3 (1.34%)	157 (2.32%)	0.897533	0.997157	ko03010
Leukocyte transendothelial migration	1 (0.45%)	68 (1.00%)	0.899699	0.997157	ko04670
ECM-receptor interaction	2 (0.89%)	118 (1.74%)	0.906909	0.997157	ko04512
RNA polymerase	2 (0.89%)	119 (1.76%)	0.909419	0.997157	ko03020
GnRH signaling pathway	1 (0.45%)	71 (1.05%)	0.909425	0.997157	ko04912
IL-17 signaling pathway	1 (0.45%)	72 (1.06%)	0.912454	0.997157	ko04657
Circadian entrainment	1 (0.45%)	72 (1.06%)	0.912454	0.997157	ko04713
Valine, leucine and isoleucine degradation	1 (0.45%)	73 (1.08%)	0.915382	0.997157	ko00280
Purine metabolism	2 (0.89%)	122 (1.80%)	0.916576	0.997157	ko00230
Relaxin signaling pathway	2 (0.89%)	123 (1.82%)	0.918842	0.997157	ko04926
Notch signaling pathway	1 (0.45%)	75 (1.11%)	0.920948	0.997157	ko04330
Aldosterone synthesis and secretion	2 (0.89%)	124 (1.83%)	0.92105	0.997157	ko04925
Adrenergic signaling in cardiomyocytes	2 (0.89%)	125 (1.85%)	0.923201	0.997157	ko04261
Endocytosis	5 (2.23%)	254 (3.75%)	0.928467	0.997157	ko04144
Insulin secretion	1 (0.45%)	81 (1.20%)	0.935553	0.997157	ko04911
Wnt signaling pathway	2 (0.89%)	132 (1.95%)	0.936789	0.997157	ko04310
MAPK signaling pathway	3 (1.34%)	182 (2.69%)	0.944242	0.997157	ko04010
Thyroid hormone signaling pathway	3 (1.34%)	183 (2.70%)	0.945619	0.997157	ko04919
Parathyroid hormone synthesis, secretion and action	1 (0.45%)	87 (1.29%)	0.947469	0.997157	ko04928
Basal transcription factors	1 (0.45%)	89 (1.31%)	0.950932	0.997157	ko03022
mTOR signaling pathway	2 (0.89%)	144 (2.13%)	0.954948	0.997157	ko04150
Spliceosome	3 (1.34%)	201 (2.97%)	0.965594	0.997157	ko03040
Phospholipase D signaling pathway	1 (0.45%)	104 (1.54%)	0.970596	0.997157	ko04072
RNA degradation	1 (0.45%)	107 (1.58%)	0.973462	0.997157	ko03018
Neuroactive ligand-receptor interaction	2 (0.89%)	163 (2.41%)	0.973939	0.997157	ko04080

Apelin signaling pathway	1 (0.45%)	111 (1.64%)	0.976856	0.997157	ko04371
Oxytocin signaling pathway	1 (0.45%)	118 (1.74%)	0.981787	0.997157	ko04921
Amino sugar and nucleotide sugar metabolism	1 (0.45%)	126 (1.86%)	0.986155	0.997157	ko00520
Thermogenesis	3 (1.34%)	239 (3.53%)	0.987451	0.997157	ko04714
Tight junction	1 (0.45%)	148 (2.19%)	0.993497	0.997157	ko04530
Longevity regulating pathway-worm	1 (0.45%)	172 (2.54%)	0.997157	0.997157	ko04212

Table S6. Summary of the KEGG enrichment analysis of the common DEGs between the Lab_SS-vs-R_cfm group and the Lab_SS-vs-R_bft group of *T. urticae*

Pathway	DEGs with pathway annotation (34)	All genes with pathway annotation (6770)	P-value	Q-value	Pathway ID
Salivary secretion	4 (11.76%)	120 (1.77%)	0.002887	0.087766	ko04970
Antigen processing and presentation	4 (11.76%)	124 (1.83%)	0.003251	0.087766	ko04612
One carbon pool by folate	2 (5.88%)	22 (0.32%)	0.005311	0.095591	ko00670
Lysosome	7 (20.59%)	464 (6.85%)	0.007219	0.097454	ko04142
Fc gamma R-mediated phagocytosis	3 (8.82%)	89 (1.31%)	0.009793	0.105765	ko04666
Steroid biosynthesis	2 (5.88%)	62 (0.92%)	0.038372	0.345350	ko00100
Regulation of actin cytoskeleton	3 (8.82%)	169 (2.50%)	0.051979	0.400980	ko04810
Autophagy - animal	3 (8.82%)	197 (2.91%)	0.075138	0.473341	ko04140
Adherens junction	2 (5.88%)	93 (1.37%)	0.078890	0.473341	ko04520
Ribosome biogenesis in eukaryotes	2 (5.88%)	107 (1.58%)	0.100189	0.541020	ko03008
Pentose phosphate pathway	1 (2.94%)	42 (0.62%)	0.191122	0.634548	ko00030
Lysine degradation	2 (5.88%)	163 (2.41%)	0.196898	0.634548	ko00310
Apoptosis	2 (5.88%)	163 (2.41%)	0.196898	0.634548	ko04210
Glycine, serine and threonine metabolism	1 (2.94%)	46 (0.68%)	0.207352	0.634548	ko00260
Autophagy - other	1 (2.94%)	46 (0.68%)	0.207352	0.634548	ko04136
Cholesterol metabolism	2 (5.88%)	169 (2.50%)	0.207941	0.634548	ko04979
Folate biosynthesis	1 (2.94%)	47 (0.69%)	0.211360	0.634548	ko00790
Fructose and mannose metabolism	1 (2.94%)	48 (0.71%)	0.215349	0.634548	ko00051
Galactose metabolism	1 (2.94%)	50 (0.74%)	0.223267	0.634548	ko00052
Toll and Imd signaling pathway	1 (2.94%)	70 (1.03%)	0.298300	0.653003	ko04624
Longevity regulating pathway	1 (2.94%)	75 (1.11%)	0.315930	0.653003	ko04211
Notch signaling pathway	1 (2.94%)	75 (1.11%)	0.315930	0.653003	ko04330
GABAergic synapse	1 (2.94%)	77 (1.14%)	0.322861	0.653003	ko04727
Other glycan degradation	1 (2.94%)	79 (1.17%)	0.329724	0.653003	ko00511
Sphingolipid metabolism	1 (2.94%)	83 (1.23%)	0.343247	0.653003	ko00600
Renin secretion	1 (2.94%)	90 (1.33%)	0.366278	0.653003	ko04924
Estrogen signaling pathway	1 (2.94%)	95 (1.40%)	0.382247	0.653003	ko04915
Glycerolipid metabolism	1 (2.94%)	99 (1.46%)	0.394741	0.653003	ko00561

Linoleic acid metabolism	1 (2.94%)	100 (1.48%)	0.397826	0.653003	ko00591
RNA degradation	1 (2.94%)	107 (1.58%)	0.418996	0.653003	ko03018
Ovarian steroidogenesis	1 (2.94%)	112 (1.65%)	0.433674	0.653003	ko04913
ECM-receptor interaction	1 (2.94%)	118 (1.74%)	0.450812	0.653003	ko04512
Arachidonic acid metabolism	1 (2.94%)	119 (1.76%)	0.453619	0.653003	ko00590
RNA polymerase	1 (2.94%)	119 (1.76%)	0.453619	0.653003	ko03020
Axon guidance	1 (2.94%)	119 (1.76%)	0.453619	0.653003	ko04360
mRNA surveillance pathway	1 (2.94%)	120 (1.77%)	0.456412	0.653003	ko03015
NOD-like receptor signaling pathway	1 (2.94%)	120 (1.77%)	0.456412	0.653003	ko04621
Retrograde endocannabinoid signaling	1 (2.94%)	128 (1.89%)	0.478264	0.653003	ko04723
cGMP-PKG signaling pathway	1 (2.94%)	134 (1.98%)	0.494090	0.653003	ko04022
Pancreatic secretion	1 (2.94%)	135 (1.99%)	0.496682	0.653003	ko04972
Pentose and glucuronate interconversions	1 (2.94%)	136 (2.01%)	0.499262	0.653003	ko00040
Serotonergic synapse	1 (2.94%)	151 (2.23%)	0.536444	0.653003	ko04726
Inflammatory mediator regulation of TRP channels	1 (2.94%)	153 (2.26%)	0.541194	0.653003	ko04750
Rap1 signaling pathway	1 (2.94%)	159 (2.35%)	0.555164	0.653003	ko04015
Retinol metabolism	1 (2.94%)	162 (2.39%)	0.561993	0.653003	ko00830
Neuroactive ligand-receptor interaction	1 (2.94%)	163 (2.41%)	0.564247	0.653003	ko04080
Calcium signaling pathway	1 (2.94%)	168 (2.48%)	0.575347	0.653003	ko04020
Longevity regulating pathway - worm	1 (2.94%)	172 (2.54%)	0.584030	0.653003	ko04212
Phagosome	1 (2.94%)	176 (2.60%)	0.592540	0.653003	ko04145
MAPK signaling pathway	1 (2.94%)	182 (2.69%)	0.604988	0.653387	ko04010
Spliceosome	1 (2.94%)	201 (2.97%)	0.642023	0.679789	ko03040
Focal adhesion	1 (2.94%)	229 (3.38%)	0.690527	0.717086	ko04510
PI3K-Akt signaling pathway	1 (2.94%)	245 (3.62%)	0.715313	0.728810	ko04151

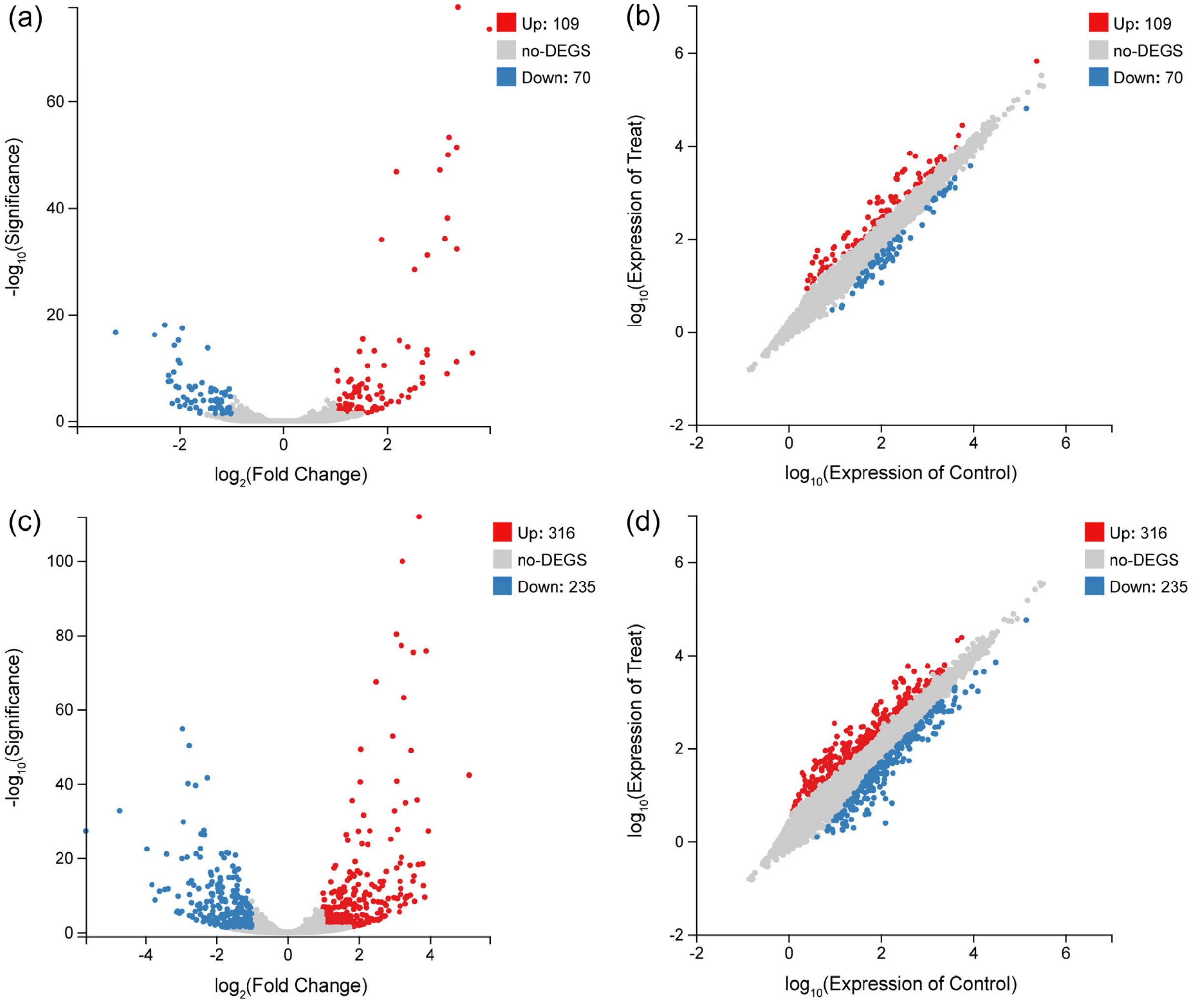

Figure S1. RNA-Seq analysis to identify genes differentially expressed in mites of Lab_SS-vs-R_cfm group and Lab_SS-vs-R_bft group. (a, b) Volcano and scatter plot for the Lab_SS-vs-R_cfm group; (c, d) Volcano and scatter for the Lab_SS-vs-R_bft group.

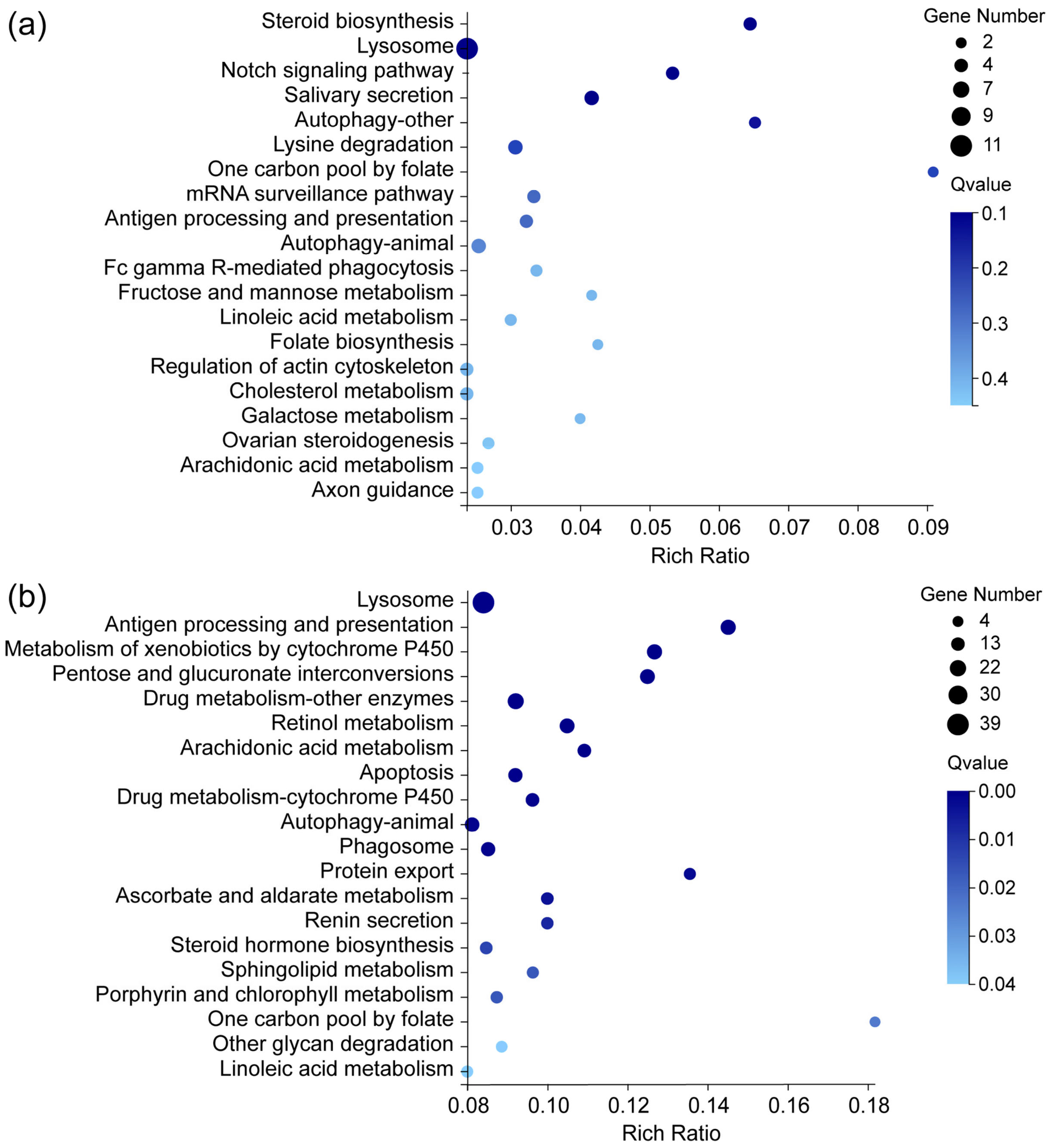

Figure S2. The top 20 biological metabolic pathways obtained from the cyflumetofen- (R_cfm; a) and bifenthrin-resistant (R_bft; b) strains of *T. urticae* in the KEGG database.

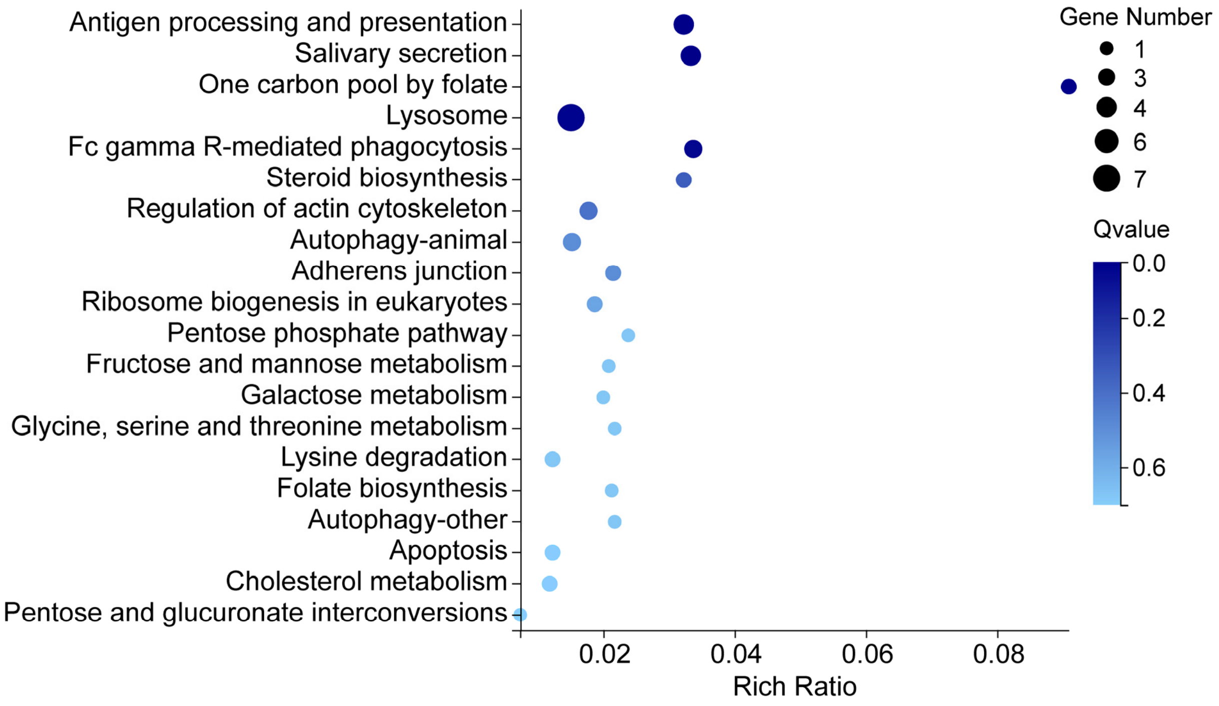

Figure S3. KEGG analysis of the common differentially expressed genes to the Lab_SS-vs-R_cfm group and the Lab_SS-vs-R_bft group.
